# Structural engineering, BSA binding and computational analysis of isonipacotate based enzyme inhibitors containing 1,2,4-triazole

**DOI:** 10.1371/journal.pone.0337642

**Published:** 2026-01-07

**Authors:** Naeem A. Virk, Aziz-ur-Rehman, Javed Iqbal, Tahir Ali Chohan, Abdullah R. Alzahrani, Talha Jawaid, Zia Ur Rehman, Abida Khan

**Affiliations:** 1 Department of Chemistry, Government College University, Lahore, Pakistan; 2 Department of Chemistry, University of Sahiwal, Sahiwal, Pakistan; 3 Institute of Pharmaceutical Sciences, University of Veterinary and Animal Sciences, Lahore, Punjab, Pakistan; 4 Department of Pharmacology and Toxicology, Faculty of Medicine, Umm Al-Qura University, Al-Abidiyah, Makkah, Saudi Arabia; 5 Department of Pharmacology, College of Medicine, Imam Mohammad Ibn Saud Islamic University (IMSIU), Riyadh, Saudi Arabia; 6 Health Research Centre, Jazan University, Jazan, Saudi Arabia; 7 Department of Pharmaceutical Chemistry and Pharmacognosy, Faculty of Pharmacy, Jazan University, Jazan, Saudi Arabia; 8 Center For Health Research, Northern Border University, Arar, Saudi Arabia; Kafrelsheikh University Faculty of Pharmacy, EGYPT

## Abstract

Synthetic chemistry facing little difficulties in C–C, C–S, and C–N double bonds activation is a very long-standing challenge. The current studies based on the development of C–N double and single bond, N–N bond and C–S in the trizole and azinane merged rings into single unit. The targeted compounds were obtained through metal free, microwave assisted and conventional techniques. The present work covers sulfonamide, hydrazide synthesis, 1,2,4-triazole, and thioether synthesis to produce piperidine-based triazole analogues (**7a-j**). An array of electrophiles (**6a-j**) was treated with 1,2,4-triazole (**5**) under conventional (59–72% yield) and microwave assisted (87–95% yield) protocols to acquire the targeted molecules (**7a-j**). The proton and carbon NMR, and IR techniques were used for structural characterization. The analogues (**7a-j**) were screened against BChE, α-Glucosidase, 15-LOX, and AChE in search for leads compounds. The most active analogues found through IC_50_ values in μM against AChE were **7h** (1.29 ± 1.24), **7c** (2.58 ± 1.32) and **7i** (5.62 ± 1.35) compared with the standard eserine (0.19 ± 0.05). Compound **7i** (2.24 ± 1.80) of the synthesized analogue was found most active against BChE. Compounds against 15-LOX were **7i** (2.07 ± 1.17) and **7e** (2.12 ± 0.37) found even better than the standard quercetin (2.34 ± 0.35). Most of the synthesized analogues (**7a**, **7d-7j**) showed very excellent potential strength against α-Glucosidase even efficient than reference drug acarbose (38.25 ± 0.12) presented in Table 3. The *in vitro* results were confirmed by molecular docking investigations of the active ligands with the targeted enzymes. Bovine serum albumin (BSA) binding studies displayed how the ligands interacted with BSA. The data altogether predicts these molecules as leads in search for cholinesterase (**7h, 7c, 7i**) and 15-LOX (**7i**) enzymes. Further *in vivo* work is continued on targeted derivatives of 1,2,4- triazoles as inhibitors of therapeutically important enzymes.

## 1 Introduction

The energy utilization with digitally controlled systems during any chemical reaction has been a common practice exercised by synthetic chemist [1]. The carelessness of energy utilization during a reaction may destroy the more robust reaction system. So, it is very important to choose an appropriate energy source. Microwave irradiation has assisted excellently in providing controlled energy system [2–5]. It is also evident from our earlier reported research that this methodology offered pure and high yield of product within short period of time [6]. It is reported that the use of microwave assisted protocol for controlled and pure synthesis on small and large scale has been applied [7–10]. Some of the best advantages of microwave irradiation are environment friendly feature, controlled and reduced consumption of energy [11]. The reactions involving the interaction of a variety of components advanced the synthesis of organic framework with multiple functionalities [12–16]. Among various isomers of triazoles, 1,2,4-triazole and its analogues proved to be significant due to its potential in different fields like agriculture, pharmaceutical, material science and biochemistry. Literature citations have shown that the structural motif combination of triazole and piperidine ring leads to thriving field of conjugate systems with broad spectrum of biological applications [17–19]. The literature survey justified the importance of triazole based hybrids possessing antiviral, antifungal, antibacterial, anti-inflammatory, anticonvulsant and anticancer activities [20–25].

Memory loss is a symptom of dementia, a neurological condition. It is linked with memory loss, diminished thinking and cognitive ability, depression, and difficulty carrying out daily tasks [26]. The complex clinical symptoms dementia are brought on by chronic oxidative strain, breakdown of mitochondria, extrinsic beta-amyloid production, and intracellular neurofibrillary network accumulation. According to cholinergic hypothesis, the two acetylcholinesterase (AChE) and butyrylcholinesterase (BChE) enzymes are controlling cholinergic neurotransmission throughout the body though BChE is also involved in other functions [27]. Acetylcholine is the major important neurotransmitters in brain synapses, if it is hydrolyzed due to over expression of AChE or BChE, impairment in neuronal signaling will result in neurodegenerative disease like AD. Therefore, the inhibition of these two enzymes is central in the treatment of AD. Although several drugs are marketed but severe side effects warrant search for new inhibitors as leads against AD and related disorders [28]. The monitoring of BChE levels is also crucial in AD, especially in the late phases when levels of BChE increase. When it comes to type 2 diabetes (T2DM), maintaining levels of BChE is also essential [29]. Therefore, the inhibitory studies of both these enzymes are crucial in search for anti-cholinesterase agents. Inflammation is related with almost all forms of ailments including asthma, gastrointestinal ulcers, neuronal disorders and several types of cancers. Cyclooxygenase (COX) and lipoxygenase (LOX) pathways utilize arachidonic acid as substrate and produce lipid derived biologically active mediators including prostaglandins, thromboxane, prostacyclin’s, leukotrienes which are responsible for inflammation through various signaling cascades [30–32]. NSAIDs like ibuprofen and mefenamic acid, diclofenac acid are used globally as anti-inflammatory agents which act by inhibiting the COX pathway [33]. Zileuton is the only marketed LOX inhibitory drug to treat asthma. The inhibition of LOX pathway is therefore an alternative to treat inflammation [34]. A wide range of naturally occurring floura (*Cornus officinalis, Binzi cultivars, ‘Xiangbinzi)* used as traditional medicine have heterocyclic systems which guarantee the biological potential of heterocyclic systems [35–37]. With the aim to synthesize 1,2,4-triazole hybrids after submerging this nucleus with some other bioactive functionalities through comparative synthetic approaches assisted with *in vitro* and *in silico* methodologies is the part of current studies. This fruitful effort introduced new compounds with purity and considerable yield which possessed good to poor inhibition potentials against 15-LOX, AChE, α-Glucosidase, and BChE.

## 2 Results and discussions

**Fig 1:**
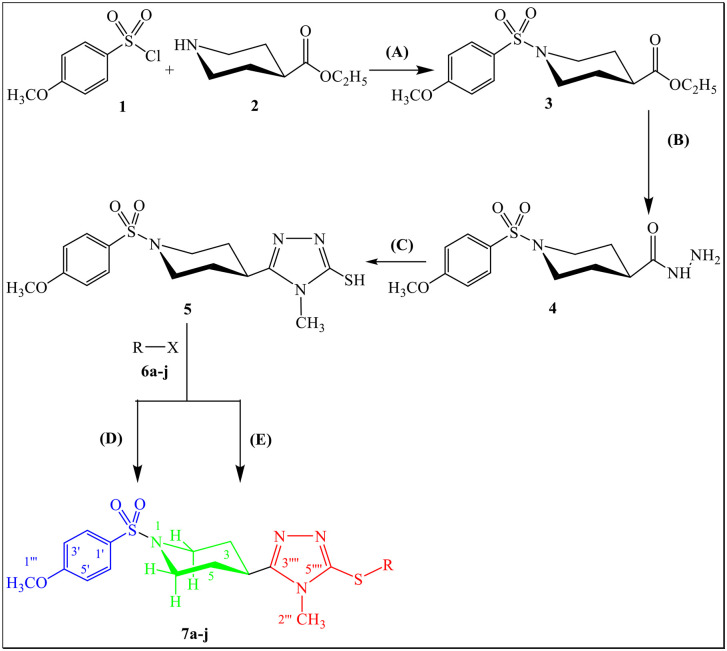
Synthesis scheme of 2,5-disubstituted-1,2,4-triazole (7a-j). Reagents and conditions: (A) H_2_O, 5.5% Na_2_CO_3_ soln., pH = 8-10, Stirr at RT (B) NH_2_NH_2_.H_2_O, MeOH, Reflux (C) i. MeNCS, EtOH, Reflux, ii. 9% KOH, Reflux (D) DMF, NaH, Stirring at RT (E) DMF, NaH, Microwaves.

**Fig 2:**
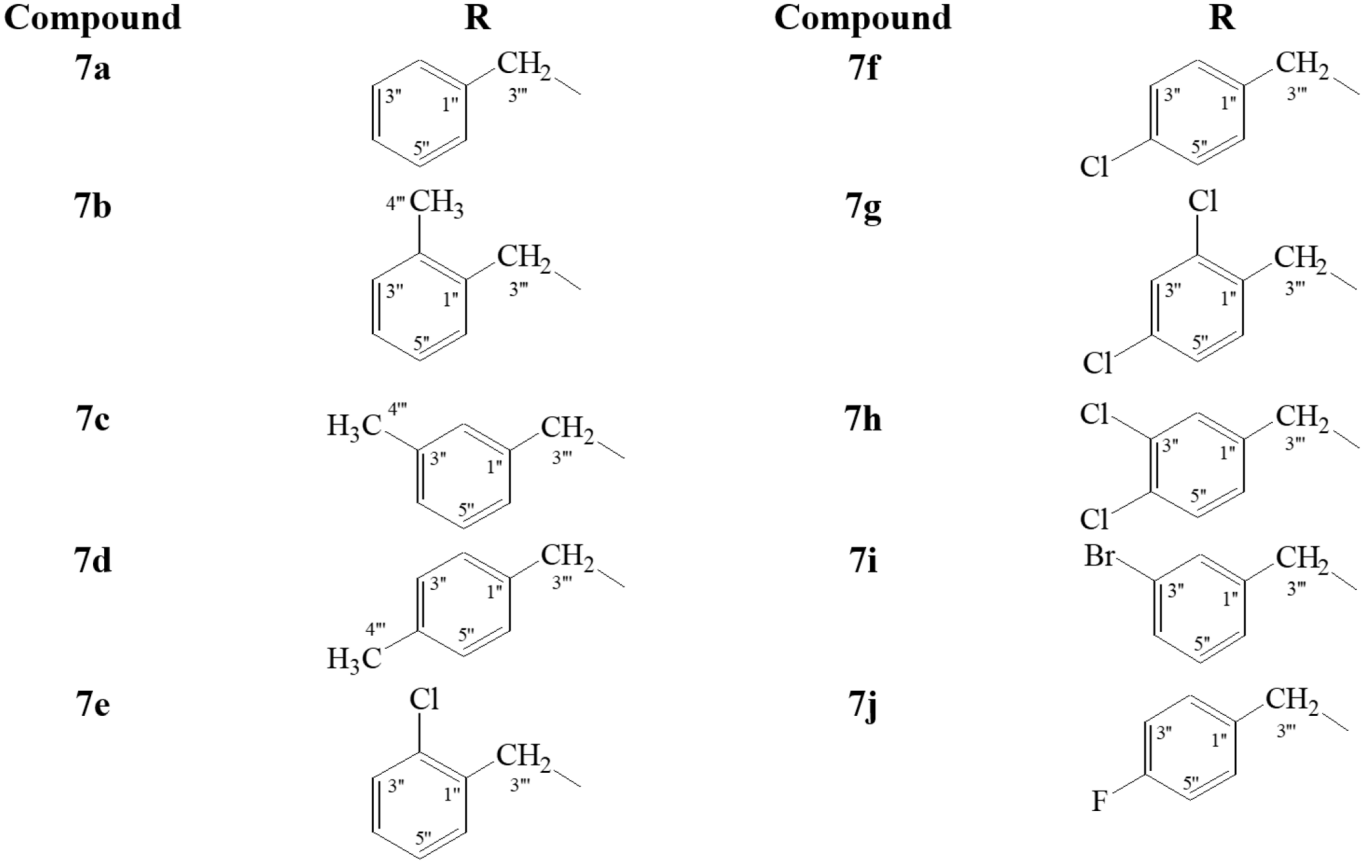
Different aralkyl groups involved in the synthesis of compounds (7a-j).

**7a-j** Compounds were manufactured utilizing the methodology given in Fig 1 with the varying part of main molecular core of aralkyl groups (Fig 2). The two types of synthetic protocols were exercised on comparative basis in terms of synthetic time and yield for the library of compounds, **7a-j**. Among both synthetic protocols, one was conventional, and the other was microwave assisted protocol. Comparative study of both synthetic protocols justified that the microwave-based synthesis was better than the conventional one because whole intended molecules were synthesized in fractions of a minute as mentioned in Table 1. Time taken for completion of reaction was 0.53–1.13 minutes for microwave assisted methodology compared to the conventional one taking 480–960 minutes. Similarly, the purity and yield of designed compounds was maximum in microwave assisted strategy (Table 1). All the synthesized hybrids yield was 59–72% by following the conventional technique while it was 87–95% by following the microwave assisted methodology.

**Table 1:** Comparative microwave assisted and conventional synthesis.

Compounds	Reaction Time (minutes)		Yield (%)	
	Conventional	Microwave	Conventional	Microwave
7a	720	0.58	62	94
7b	480	0.53	59	90
7c	420	1.02	66	93
7d	780	1.13	60	89
7e	540	0.59	70	92
7f	900	1.04	63	91
7g	600	0.56	72	95
7h	960	1.07	66	88
7i	480	1.00	69	90
7j	540	1.01	62	87

### 2.1 Chemistry

**Fig 3:**
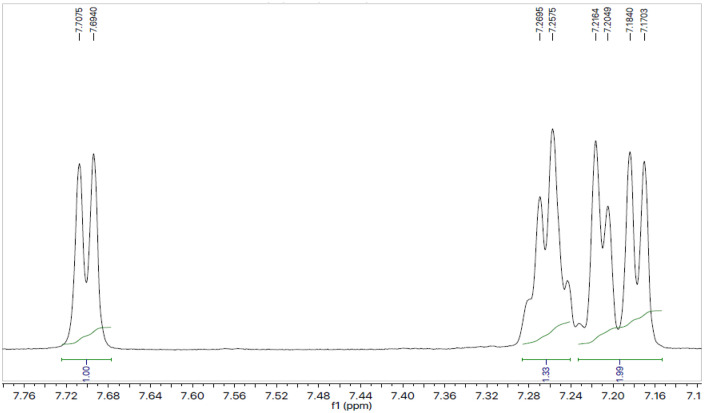
^1^H-NMR spectra of 3-benzylthio-5-{1-[(4-methoxyphenyl)sulfonyl]-4-piperidinyl}-4-methyl-4*H*-1,2,4-triazole (7a) (aromatic region).

**Fig 4:**
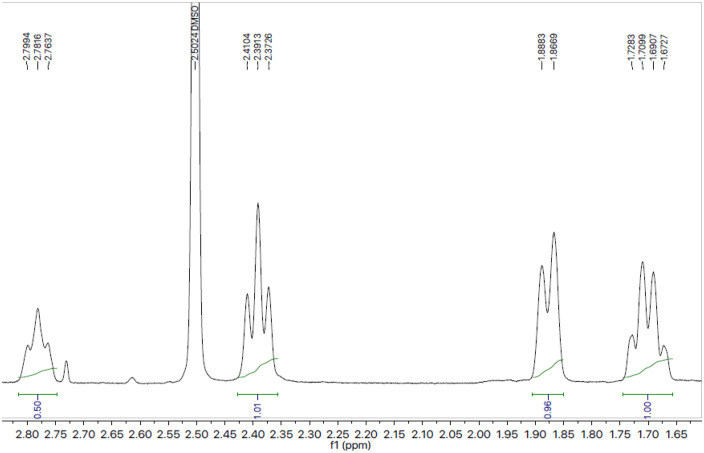
^1^H-NMR spectra (aliphatic hydrogens) of 3-benzylthio-5-{1-[(4-methoxyphenyl)sulfonyl]-4-piperidinyl}-4-methyl-4*H*-1,2,4-triazole (7a).

Utilizing compound **7a** as a point of reference, the structural elucidation was completed using a similar methodology to the rest of the synthesized chemical series. At a melting point of 142 °C, compound **7a** was produced as a granular white form. By the information obtained by spectral studies, the molecular formula and molar mass of compound **7a** were designated as C_22_H_26_N_4_O_3_S_2_ and 458.59 g/mol, respectively. The functional groups available in the selected compound **7a** were confirmed by IR studies. The various functional groups showed peaks at 2806 (Aromatic carbon hydrogen single bond), 1605 (Carbon nitrogen double bond), 1524 (Aromatic carbon carbon double bond), 1362 (methyl), 1315 (Sulphur monoxide) and 1204 (Carbon oxygen single bond) which proved to be helpful for structural justification of compound **7a**. The presence of various H-atoms, their position and numbers were justified by the spectral details obtained through H^1^-NMR spectra (Figs 3, 4) obtained by dissolving compound in deuterated chloroform. The four aromatic hydrogen atoms directly linked with sulfonyl moiety appeared as a two doublet peaks 7.69 (d, coupling constant = 8.1 Hz, 2H, 2’,6’) and 7.18 (d, coupling constant = 8.2 Hz, 2H, 3’,5’). *S*-Substituted aromatic protons were confirmed by one multiplet signal appearing at 7.26–7.25 (m, 3H, 3’‘,4’‘,5’‘) and one doublet signal at 7.21 (d, coupling constant = 6.9 Hz, 2H, 2’‘,6’‘). After confirmation of aromatic moieties, we next moved towards the aliphatic region of the compound **7a**. All of piperidine’s protons were supported by the multiplet peaks at 3.62-3.60 (m, 2H, 2e, 6e), 2.79-2.76 (m, 1H, 4), 2.41-2.37 (m, 2H, 2a, 6a), 1.88-1.86 (m, 2H, 3e, 5e) and 1.72-1.67 (m, 2H, 3a, 5a). Protons belonging to the methoxy group were verified by a singlet that emerged at 3.86 (s, 3H, 1’“). However, a singlet justified methyl group connected to the triazole ring’s nitrogen at 3.81 (s, 3H, 2’“). A singlet justified two hydrogens of CH_2_ group sandwiched between *S*-atom and an aromatic ring at 4.23 (s, 2H, 3’“).

**Fig 5:**
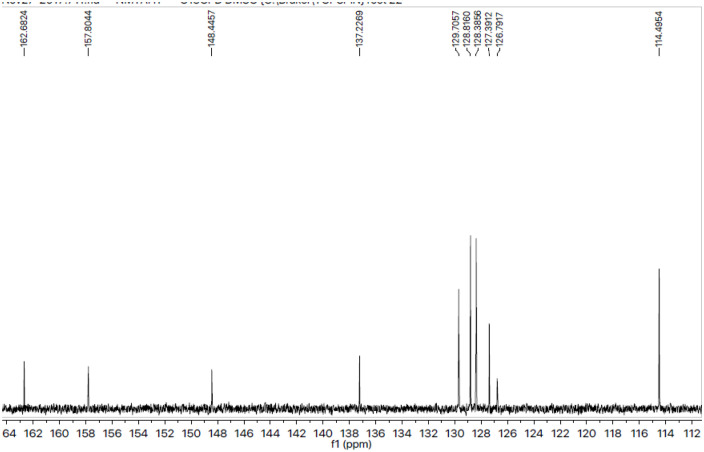
^13^C-NMR spectra of 3-benzylthio-5-{1-[(4-methoxyphenyl)sulfonyl]-4-piperidinyl}-4-methyl-4*H*-1,2,4-triazole (7a) (aromatic).

**Fig 6:**
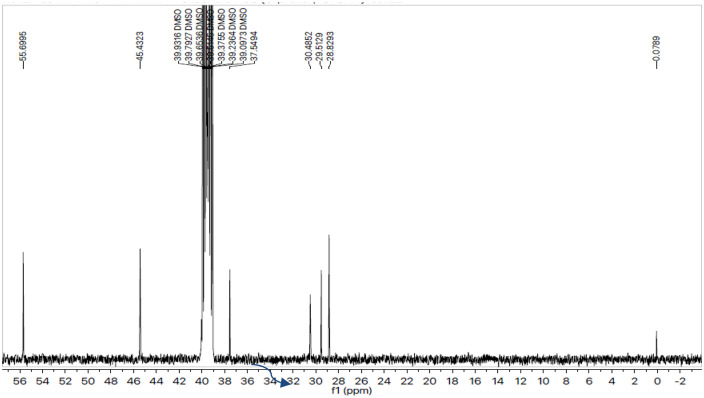
^13^C-NMR spectra of 3-benzylthio-5-{1-[(4-methoxyphenyl)sulfonyl]-4-piperidinyl}-4-methyl-4*H*-1,2,4-triazole (7a) (aliphatic).

The different types of carbon atoms constructing the molecular structure were justified by the ^13^C-NMR spectral studies (Figs 5, 6). The presence ofpeaks at 162.68 (C-4’), 157.80 (C-5’“‘), 148.44 (C-3’“‘), 137.22 (C-1’‘) and 126.79 (C-1’) confirmed the various quaternary carbons. The peaks that appeared at 129.70 (C-2’, C-6’), 114.49 (C-3’, C-5’), 128.81 (C-2’‘, C-6’‘), 128.38 (C-3’‘, C-5’‘), and 127.39 (C-4’‘) validated the methine carbons of two aromatic rings connected with sulfonyl and thio groups. The peaks at 45.43 (C-2, C-6), 30.48 (C-4), and 28.82 (C-3, C-5) confirmed the presence of piperidine’s carbon skeleton. In terms of OCH3, CH3, and CH2, the remaining aliphatic region’s carbons were found at 55.69 (C-1’“), 37.54 (C-2’“), and 37.54 (C-3’“), respectively. The detailed spectral studies helped to confirm the structural details of compound **7a,** 3-benzylthio-5-{1-[(4-methoxyphenyl)sulfonyl]-4-piperidinyl}-4-methyl-4*H*-1,2,4-triazole*.* Similarly the chemistry and structure of all compounds were confirmed using spectra given in S1-S20 Figs, in similar pattern as compounds **7a**.

### 2.2 Biological screening

#### 2.2.1 Acetyl and butrylcholinestrase inhibition studies.

**Fig 7:**
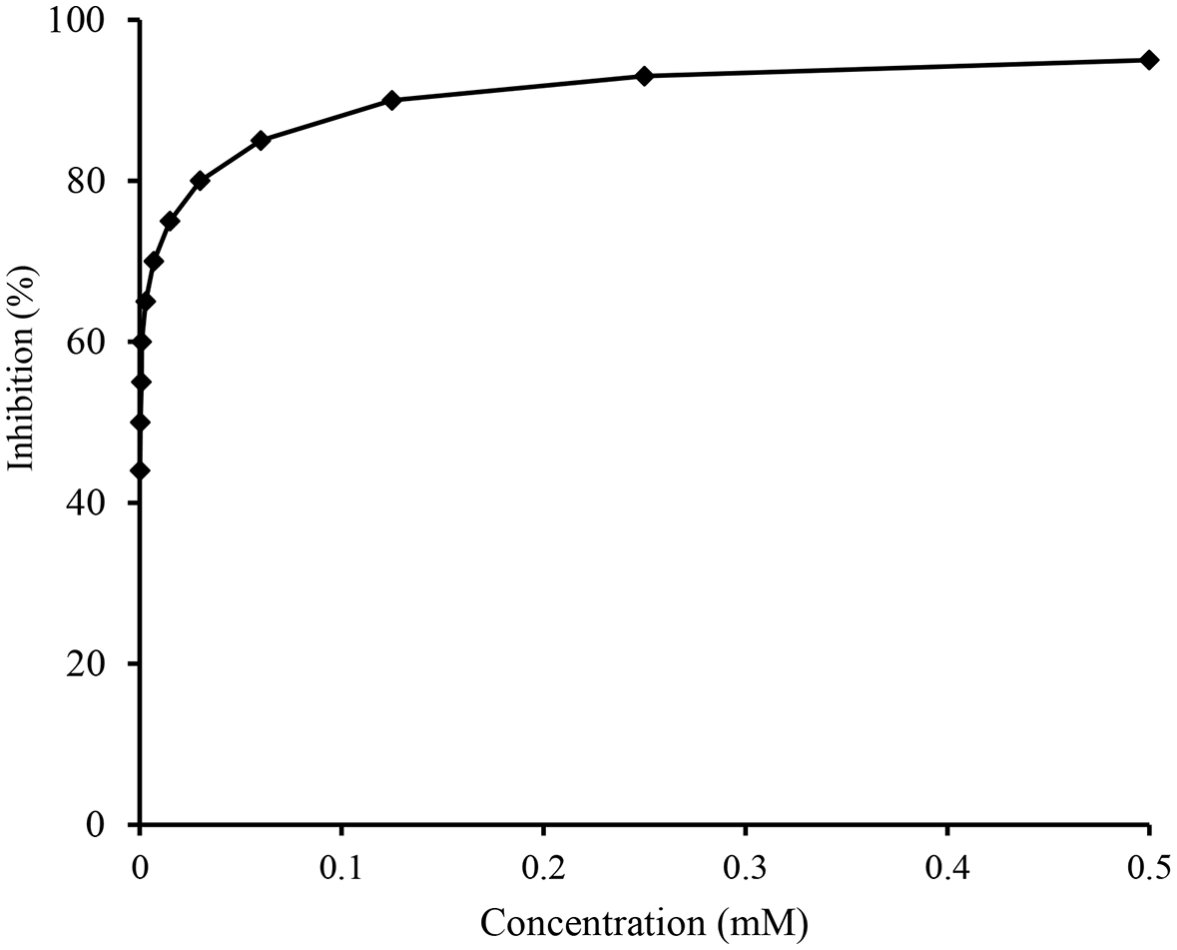
The effect of variable concentration of compound 7h on the inhibitory profile of AChE. The calculated IC_50_ value was 1.29 ± 1.24µM. The error bars are not inserted because of simplicity.

**Fig 8:**
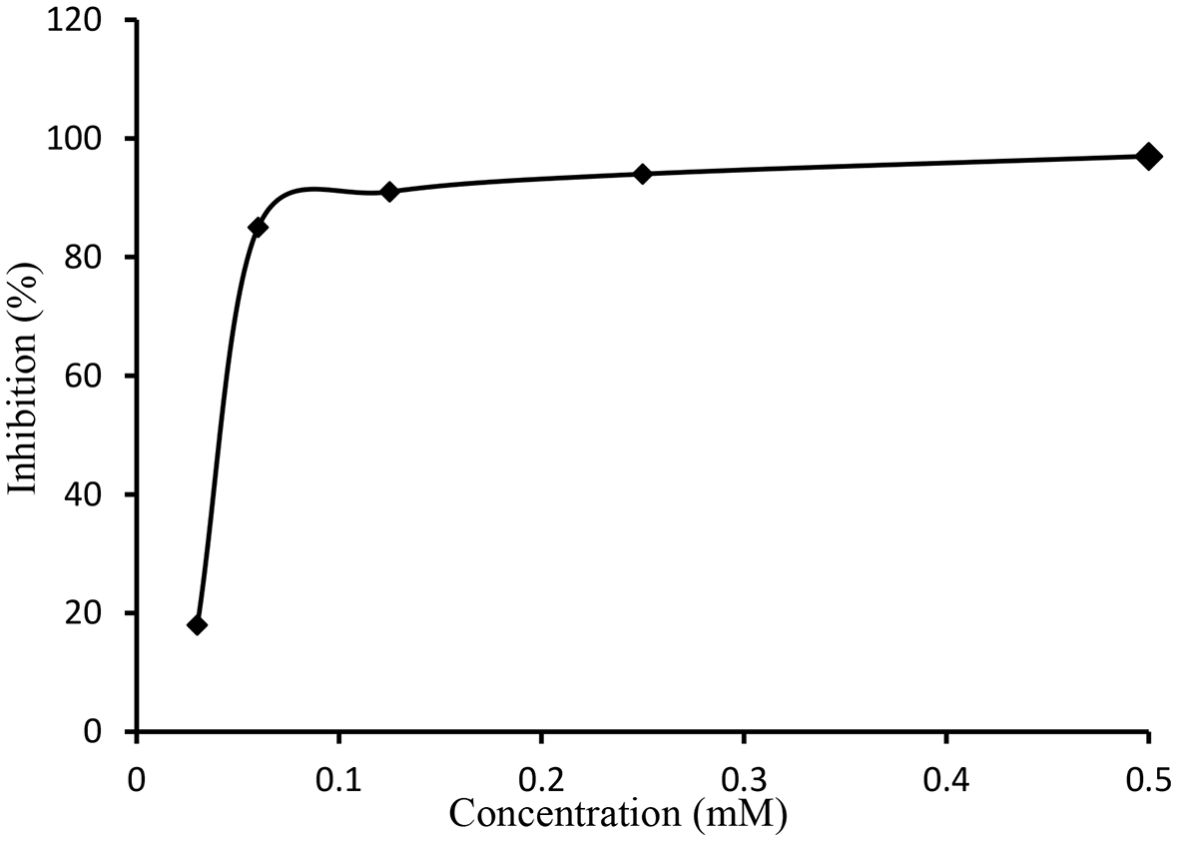
The effect of variable concentration of compound 7c on the inhibitory profile of AChE. The calculated IC_50_ value was 2.58 ± 1.32 µM. The error bars are not inserted because of simplicity.

The final products **7a-j** were screened against acetyl and butrylcholinestraseand data revealed good inhibiting activities of **7h** (IC_50_ 1.29 ± 1.24 μM), **7c** (IC_50_ 2.58 ± 1.32 μM) and **7i** (IC_50_ 5.97 ± 1.35 μM) against AChE and none of the compound was active against BChE (Table 2, Figs 7, 8). Likewise compound **7i** (IC_50_ 2.24 ± 1.80 μM) and**7e**(IC_50_ 3.92 ± 0.46 μM) showed highest potential against BChE. All compounds (**7a-7j**) discovered to be highly active with range of IC_50_ 1.29 ± 1.24–129.31 ± 1.23 μM and IC_50_ 2.24 ± 1.80–93.45 ± 1.54 μM against AChE and BChE respectively. Little SAR could be established that the highest potential shown by compound **7c** and **7h** against AChE might be due to *S*-substitution by 3-methylbenzyl and 3,4-dichlorobenzyl moieties, respectively.

**Table 2:** Enzyme inhibitory potential of compounds (7a-j) against AChE, BChE, 15-LOX and α-Glucosidase (Mean±SEM, n = 3).

Compounds	IC_50_ values (μM)			
	AChE	BChE	15-LOX	α-Glucosidase
7a	129.31 ± 1.23	93.45 ± 1.54	16.52 ± 0.32	29.45 ± 0.49
7b	41.35 ± 1.39	15.04 ± 0.32	18.42 ± 0.56	98.03 ± 0.41
7c	2.58 ± 1.32	14.57 ± 1.28	35.21 ± 1.15	91.13 ± 0.52
7d	30.15 ± 1.43	41.62 ± 1.37	12.25 ± 0.27	21.91 ± 0.55
7e	5.97 ± 1.37	3.92 ± 0.46	2.12 ± 0.37	18.57 ± 0.26
7f	12.45 ± 1.49	15.63 ± 1.41	4.63 ± 0.45	18.18 ± 0.35
7g	26.32 ± 1.36	59.32 ± 1.23	15.12 ± 0.24	35.05 ± 0.64
7h	1.29 ± 1.24	5.62 ± 1.37	7.64 ± 0.38	15.56 ± 0.52
7i	5.62 ± 1.35	2.24 ± 1.80	2.07 ± 1.17	19.61 ± 0.25
7j	27.54 ± 1.32	25.41 ± 1.55	41.02 ± 1.26	22.45 ± 0.66
Eserine	0.19 ± 0.05	0.62 ± 0.08	–	
Quercetin	–	–	2.34 ± 0.35	
Acarbose				38.25 ± 0.12

#### 2.2.2 15-LOX inhibition.

**Fig 9:**
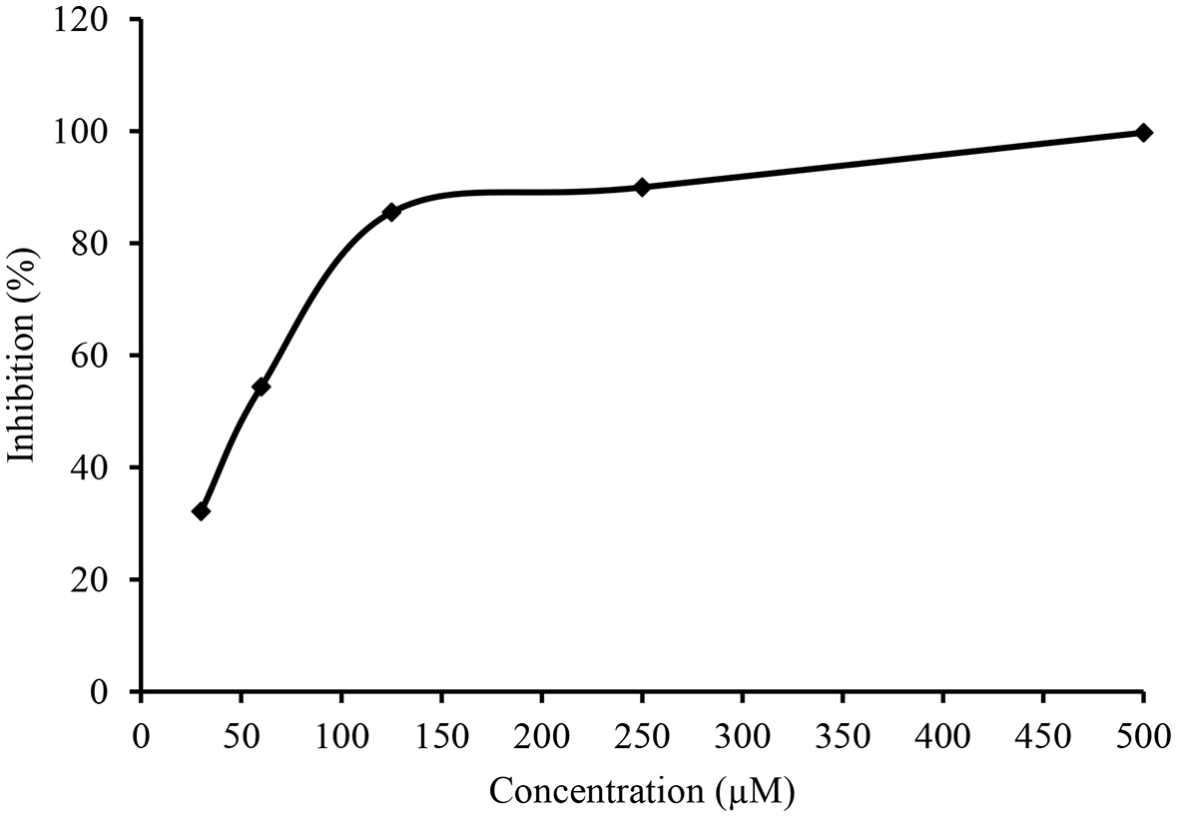
The effects of variable concentrations of compound 7i for inhibitory profile of 15-LOX. The calculated IC_50_ value was 2.07 ± 1.17 µM. The error bars are not inserted because of simplicity.

**Fig 10:**
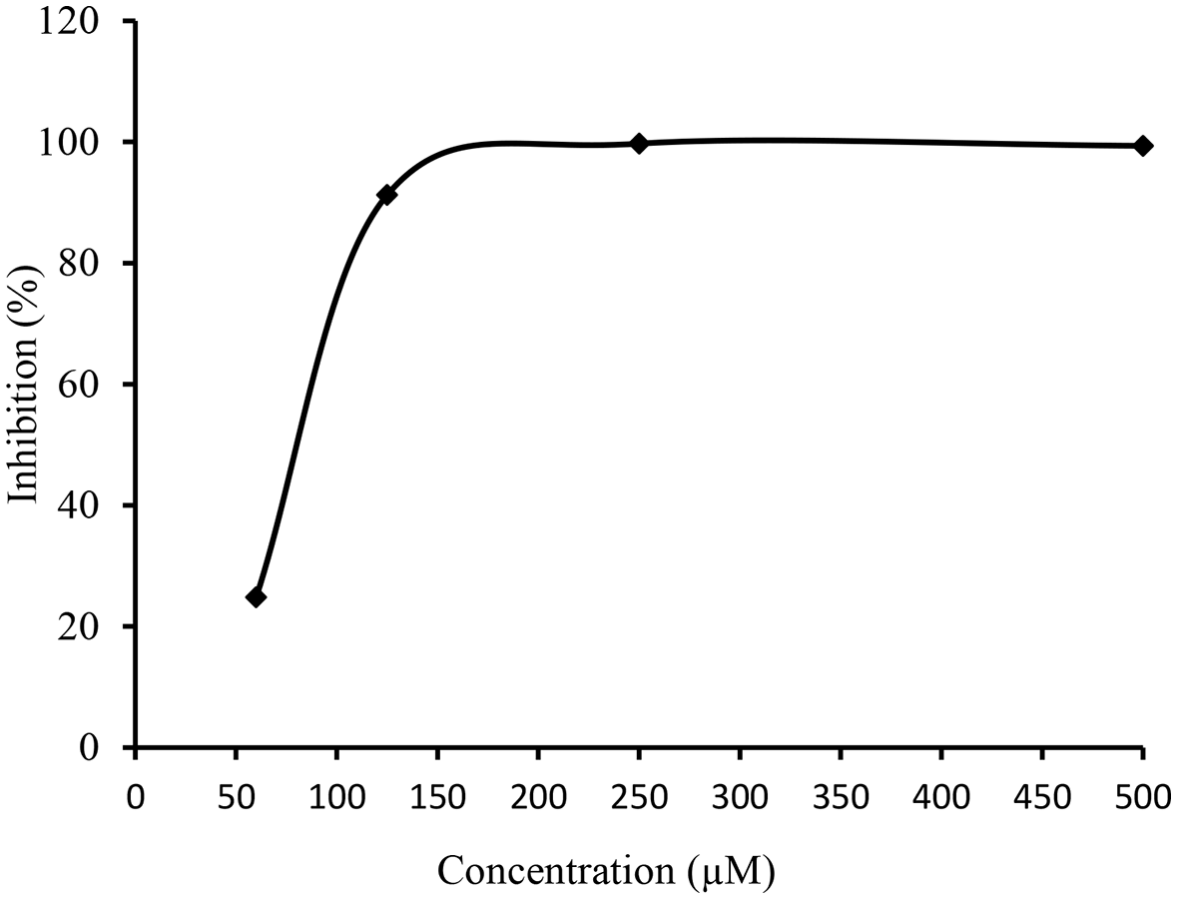
The effect of variable concentration of compound 7e on the inhibitory profile of 15-LOX. The calculated IC_50_ value was 2.12 ± 0.37 µM. The error bars are not inserted because of simplicity.

The mentioned compounds (**7a-j)** were screened against 15-LOX (Table 2) and the findings revealed that **7i** (IC_50_ 2.07 ± 1.17 μM) and **7e** (IC_50_ 2.12 ± 0.37 μM) were most active while other compounds displayed good to moderate inhibitory profiles (Figs 9, 10). Little SAR could be established, and it is speculated the good inhibitory potential for **7e** and **7i** might be due to the presence of 2-chlorobenzyl and 3-bromobenzyl moieties on the basic pharmacophore.

### 2.3 Molecular docking studies

**Fig 11:**
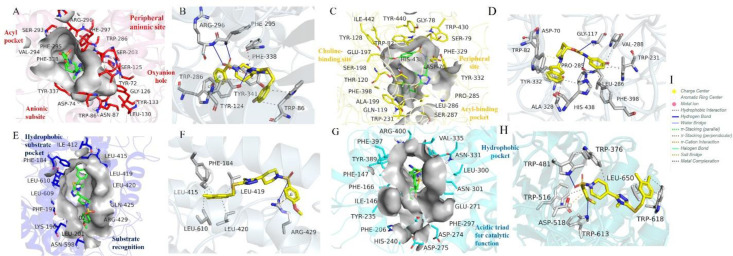
Binding pocket visualization and docking interaction analysis of selected ligands with (A–B) AChE, (C–D) BChE, (E–F) 15-LOX, and (G–H) α-glucosidase. Panels A, C, E, and G highlight the structural features of the active sites, including catalytic residues, sub-pockets, and substrate recognition motifs. Panels B, D, F, and H present PLIP-derived interaction maps showing hydrogen bonds, hydrophobic contacts, π–π stacking, and other stabilizing interactions that govern ligand binding.

The binding affinities of compounds **7a–7j** were evaluated against AChE, BChE, 15-LOX, and α-glucosidase using eserine (AChE/BChE), quercetin (15-LOX), and acarbose (α-glucosidase) as reference inhibitors. The standards yielded docking scores of eserine −8.029 (AChE) and −8.125 (BChE), quercetin −6.045 (15-LOX), and acarbose −6.443 (α-glucosidase). The test compounds displayed the following score ranges: AChE, −8.580 to −9.580 kcal·mol ⁻ ¹; BChE, −5.481 to −9.678 kcal·mol ⁻ ¹; 15-LOX, −6.123 to −7.093 kcal·mol ⁻ ¹; and α-glucosidase, −5.606 to −6.726 kcal·mol ⁻ ¹. Among them, compound **7i** exhibited the strongest binding to AChE (−9.580 kcal·mol ⁻ ¹) and BChE (−9.678 kcal·mol ⁻ ¹), while compound **7h** showed the best affinities for 15-LOX (−7.093 kcal·mol ⁻ ¹) and α-glucosidase (−6.726 kcal·mol ⁻ ¹). These results are summarized in Table 3. The PLIP-generated high-resolution interaction fingerprints (Fig 11) provide structural insights that rationalize these relative affinities; selected complexes are discussed below.

**Table 3:** Docking scores of compounds (7a-7j) against ACHE, BCHE, 15-LOX, α-glucosidase enzyme.

Sr.no	Protein	ACHE	BCHE	15-LOX	Glucosidase
	Standard	Eserine	Eserine	Quercetin	Acarbose
	Docking scores	-8.029	-8.125	-6.045	-6.443
	Compounds				
1	7a	-8.388	-8.277	-6.668	-6.124
2	7b	-5.208	-5.481	-6.123	-5.606
3	7c	-9.450	-9.103	-6.856	-5.555
4	7d	-8.584	-8.234	-6.222	-6.021
5	7e	-8.542	-8.432	-6.040	-5.992
6	7f	-8.584	-8.542	-6.322	-5.813
7	7g	-8.848	-8.763	-6.481	-6.123
8	7h	-9.384	-9.568	-7.093	-6.726
9	7i	-9.580	-9.678	-6.921	-6.625
10	7j	-8.682	-8.612	-6.682	-5.707

As shown in Fig 11A, the AChE binding pocket is defined by a narrow catalytic gorge containing the catalytic triad Ser203, His447, and Glu334, along with critical residues Trp86, Tyr124, Phe295, Phe297, and Trp286 that form the anionic and acyl subsites. Peripheral anionic site residues Y337 and Y341 further assist in substrate recognition and ligand stabilization [38]. Compound **7i**, which displayed the highest affinity (−9.580 kcal·mol ⁻ ¹), is accommodated within this aromatic-rich cavity. The PLIP analysis (Fig 11B) reveals hydrogen bonds with Tyr124 and Arg296, π–π stacking against Trp286, a π–cation interaction with Tyr341, and hydrophobic contacts with Phe338 and Trp86. These multipoint interactions anchor the ligand firmly within the catalytic gorge, explaining its superior docking performance relative to eserine.

In Fig 11C, the binding pocket of BChE is illustrated. Unlike AChE, BChE possesses a broader and more solvent-exposed gorge, with the catalytic triad formed by Ser198, His438, and Glu325. Key residues include Trp82 and Tyr332 (choline-binding site), and hydrophobic residues such as Trp231, Leu286, Val288, and Phe398 that line the acyl pocket. The oxyanion hole is constituted by Gly116, Gly117, and Ala199, while residues Asp70, Trp430, and Tyr440 contribute to peripheral stabilization [39]. Compound **7i** achieved the strongest BChE affinity (−9.678 kcal·mol ⁻ ¹), surpassing eserine (−8.125 kcal·mol ⁻ ¹). The PLIP interaction map (Fig 11D) confirms this adaptability: **7i** formed hydrogen bonds with Gly117 and Pro285, while extensive hydrophobic contacts with Trp231, Val288, Leu286, Phe398, Ala328, and Tyr332 reinforced the binding. This interaction pattern suggests that BChE ligands rely predominantly on hydrophobic stabilization, with hydrogen bonds assisting in ligand orientation.

The 15-LOX binding site (Fig 11E) is characterized by a long hydrophobic channel enclosing the catalytic non-heme iron. The catalytic site includes iron-coordinating residues His373, His378, His553, and Ile676, while hydrophobic residues Phe184, Leu415, Leu420, and Leu610 line the substrate channel. Arg429 serves as a crucial polar anchor, aiding ligand positioning [40]. Compound **7h** demonstrated the best binding score (−7.093 kcal·mol ⁻ ¹), outperforming quercetin (−6.045 kcal·mol ⁻ ¹). As depicted in Fig 11F, 7h established hydrogen bonding and π–cation interactions with Arg429, supplemented by hydrophobic contacts with Phe184, Leu415, Leu610, and Leu420. These interactions highlight the dual role of Arg429 as both a stabilizer and anchor, while the hydrophobic tunnel provides confinement, ensuring a snug ligand fit consistent with its superior docking score.

The α-glucosidase binding site (Fig 11G) comprises a catalytic acidic cluster (Asp274, Asp275, Glu271) surrounded by aromatic residues including Trp376, Trp481, Trp516, Trp613, and Trp618, forming a characteristic aromatic belt. Residue Leu650 further contributes to van der Waals stabilization. Compound **7h**, the best binder (−6.726 kcal·mol ⁻ ¹), surpassed acarbose (−6.443 kcal·mol ⁻ ¹). The PLIP interaction profile (Fig 11H) shows hydrogen bonds with Trp481 and Trp613, extensive π–π stacking with Trp376, Trp516, and Trp618, and hydrophobic stabilization with Leu650. These interactions create an “aromatic clamp” that secures the ligand near the catalytic residues, explaining its favorable score.

Overall, the PLIP interaction analysis clarifies the selectivity trends: compound **7i** excels in AChE and BChE binding by combining directional hydrogen bonds with multiple π interactions in their catalytic gorges, whereas compound **7h** exploits Arg429-mediated interactions in 15-LOX and a tryptophan-rich aromatic environment in α-glucosidase. The high-resolution interaction maps (Fig 11A–H) therefore, not only corroborate the docking score trends in Table 3 but also provide a structural rationale that informed subsequent SIFt analysis, DFT interpretation, and ADMET profiling.

### 2.4 Structural interaction fingerprinting analysis

**Fig 12:**
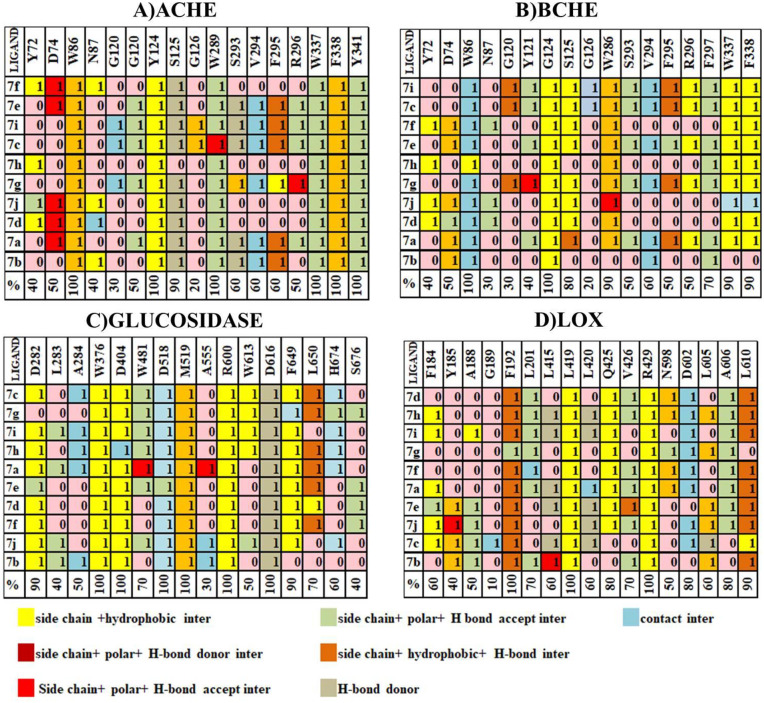
SIFt Analysis: (A-B-C-D) Protein-ligand interaction fingerprints for modelling compounds within a 4.0Å radius of interacting residues. Residue presence indicated as 1, absence as 0, and colored by hydrophobic, hydrogen bond donor, and acceptor properties.

(Fig 12A) reveals that key residues within the ligand binding cavity of ACHE include Tyr72, Asp74, Trp86, Asn87, Gly120, Gly121, Tyr124, Ser125, Gly126, Trp289, Ser293, Val294, Phe295, Arg296, Trp337, Phe338, and Tyr341. Most compounds engage in consistent interactions with the residues, namely, Trp86, Tyr124, Ser125, Trp289, Trp337, Phe338, and Tyr341, and interestingly, these residues surround the binding cavity around compound **7i** as illustrated in Fig 12A, making these residues pivotal in the binding cavity of ACHE. Two of these most engaging residues are Tyr124 and Phe295, which form hydrogen bonds with the compound of the highest affinity score as represented in Fig 12A.

(Fig 12B) reveals that key residues within the ligand binding cavity of BCHE include Tyr72, Asp74, Trp86, Asn87, Gly120, Gly124, Tyr124, Ser125, Gly126, Trp289, Val294, Phe295, Arg296, Trp337, and Phe338. Most compounds engage in consistent interactions with the residues namely Trp86, Gly124, and Trp337. One of these most engaging residues is Trp86, which forms a hydrogen bond with the compound of highest affinity score as represented in Fig 12B. Least interacting residues are Asn87, Gly120, and Gly126, showing below 50% of interaction among compounds (**7a–7j**).

Similarly, Fig 12C reveals that key residues within the ligand binding cavity of α-glucosidase include Asp282, Leu283, Ala284, Trp376, Asp404, Ile401, Trp481, Trp516, Asp518, Met519, Ser523, Asn524, Phe525, Ala555, Arg600, Trp613, Gly615, Asp616, Trp618, Asp645, Phe649, Leu650, Gly651, and His671. Residues involving consistent interaction are Trp376, Asp404, Phe525, and Trp481 with binding affinities more than 70%. One of these most engaging residues is Trp481, which forms a hydrogen bond with the highest affinity score as represented in Fig 12D. Least interacting residues are Ile441, Gly651, Leu677, and Phe525 having binding affinity below 50%.

As shown in (Fig 12) assorted colors represent different types of interactions or features between molecules or within a protein structure. Here’s an explanation of each color; yellow; side chain and hydrophobic, sky blue; General contacts between molecules or within a protein, light green; side chain, polar and hydrogen bond acceptor, Gold; side chain, hydrophobic and hydrogen contact, Light grey; hydrogen donor, Maroon; side chain, polar and hydrogen bond donor, Red; side chain, polar and hydrogen bond acceptor. These colors help visually interpret complex structural data, aiding in the understanding of molecular interactions and features. In short, our SIFt analysis indicates that not only do all the ligands exhibit high structural similarity, but they also share similar electrostatic patterns.

### 2.5 Toxicological modelling and ADME profiling

The comprehensive analysis, detailed in Table 4, represents the ADME properties, physicochemical characteristics, pharmacological toxicity, mutagenesis profile, and synthetic accessibility for the compounds evaluated in our study. The ease of synthesis, quantified through synthetic accessibility scores, highlights the practicality of compound development, with most compounds achieving scores of 5 or below, suggesting feasible synthesis. Notably, the synthetic accessibility score of **7h** and **7i** stands at 6.81 and 6.80 respectively, indicating a more straightforward synthesis. Furthermore **7h** and **7i** show impressive intestinal absorption rates 96.125% and 97.125% respectively, highlighting their potential for effective gastrointestinal uptake. **7i** stands out with an absorption rate of 97.125%, indicating exceptional bioavailability. The metabolism of these compounds involves cytochrome P450 enzymes, especially 2C9, essential for the metabolic breakdown and clearance of drugs. The total clearance of **7h** is −0.004 log ml/min/kg which is equal to **7i** clearance. Majority of compounds in the library exhibit zero toxicity. These findings suggest that these compounds may possess a favorable safety profile for use in clinical trials.

**Table 4:** Pharmacokinetic properties of compounds (7a-7j).

Sr. No.	Compound label	Pharmacokinetics										Synthetic accessibility
		Absorption	Metabolism							Excretion	Toxicity	
		Intestinal absorption (Human)	CYP							Total Clearance	AMES Toxicity	
		Numeric (% absorbed)	2D6 Sub	3A4 sub	1A2 inh	2C19 Inh	2C9 inh	2D6 inh	3A4 inh	Numeric (log ml/min/kg)	Categorical (yes/no)	Numeric
1	7a	95.593	No	Yes	No	Yes	Yes	No	Yes	0.146	No	3.38
2	7b	95.65	No	Yes	No	Yes	Yes	No	Yes	0.094	No	4.13
3	7c	95.65	No	Yes	No	Yes	Yes	No	Yes	0.094	No	2.78
4	7d	96.651	No	Yes	No	Yes	Yes	No	Yes	0.089	No	1.54
5	7e	97.112	No	Yes	No	Yes	Yes	No	Yes	0.078	No	2.49
6	7f	95.531	No	Yes	No	Yes	Yes	No	Yes	0.002	No	3.07
7	7g	95.531	No	Yes	No	Yes	Yes	No	Yes	0.142	No	2.36
8	7h	96.125	No	Yes	No	Yes	Yes	No	Yes	-0.004	No	6.81
9	7i	97.125	No	Yes	No	Yes	Yes	No	Yes	-0.004	No	6.80
10	7j	96.094	No	Yes	No	Yes	Yes	No	Yes	-0.004	No	4.44

### 2.6 MESP studies and DFT

**Fig 13:**
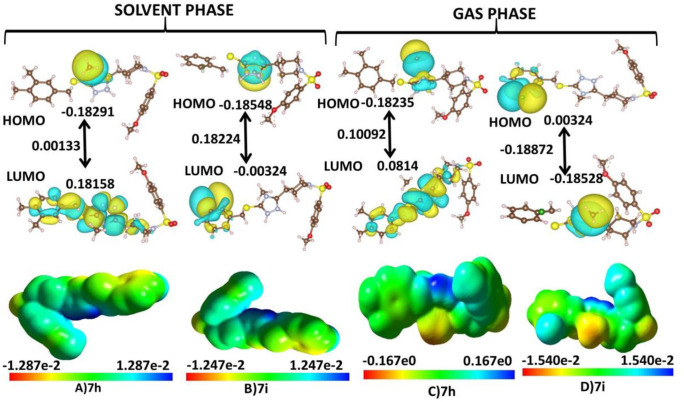
ESP structures (in solvent phases and gas phases) formed by mapping of total density over electrostatic potential, and optimized structures of 7h and 7i. Calculated HOMO and LUMO orbitals of potent derivatives at B3LYP/SVP level of DFT calculations for **7h** and **7i**.

The Molecular Electrostatic Potential (MESP) mappings have provided a comparative analysis of the electronic characteristics of all compounds (**7i-7j**), particularly focusing on two notable compounds: **7h** and **7i**. As depicted in Fig 13, these mappings highlight the distinctive electronic properties that contribute to unique biochemical interactions with ACHE, BCHE, 15-LOX and α-glucosidase. Detailed Mulliken population analysis showed that the oxygen atom at position 15 in **7h** has an average Mulliken charge of −0.813823, indicating a highly electronegative region represented as the most intensely red area on the MESP mapping. A similar trend is noted in **7i**, where nitrogen at position 16 exhibits a Mulliken charge of −0.640, enhancing its potential for hydrogen bonding within the strategic hinge and solvent-exposed regions of ACHE, BCHE, 15-LOX and α-glucosidase. Notably, **7i** displays more neutral areas (shown in green), suggesting a propensity for hydrophobic or van der Waals interactions, whereas **7h** shows a balanced representation of both red and green areas conducive to diverse interaction types. This crucial analysis helps ascertain the reactivity of the compounds. The HOMO-LUMO energy gap, indicative of the kinetic stability of a molecule, highlights the energy difference between the highest occupied and lowest unoccupied molecular orbitals, which facilitate enhanced energy transfer within the molecule. The molecular surface plots of the HOMO and LUMO orbitals for **7h** and **7i** are presented in Fig 13. The electron-acceptor capability of an inhibitor is reflected by its LUMO value, whereas the HOMO value impacts its electron-donating capacity. Table 5 summarizes a comprehensive overview of the computed quantum chemical descriptors for these compounds in aqueous conditions. DFT calculations have elucidated critical molecular properties of highest affinity score compounds (**7h** and **7i**), revealing their unique electronic structures and reactivity.

**Table 5:** Parameters for DFT analysis.

Parameters for DFT analysis											
Ligand	Dipole moment (Debye)	HOMO (a.u.)	LUMO (a.u.)	Energy Gap (ΔE_Gap_)	Ionization Potential (eV)	Electron affinity (eV)	Electronegativityχ (eV)	Electrochemical potential μ (eV)	Hardness η (eV)	Softness S (eV)	Electrophilicity ω (eV)
7h	12.918	−0.18291	0.18158	0.00133	0.18291	−0.18158	0.09686	0.18548	0.08862eV	0.509	0.448
7i	17.9947	−0.18548	−0.00324	−0.8224	0.18548	0.00324	0.0057	−0.0057	0.00254	393.70	0.0064

Notably, **7h** exhibited an extraordinarily high dipole moment of 17.9947 Debye, indicating a distinct electronic profile compared to other compounds, which demonstrated relatively lower dipole moments of 12.9918 Debye. This suggests different electron-donating capabilities and interaction potentials with ACHE, BCHE, 15-LOX and α-glucosidase. The HOMO-LUMO energy gap analysis revealed that **7i** also exhibited the largest disparity, signifying enhanced reactivity and potential biological efficacy. These findings not only deepen our understanding of the molecular basis for the inhibitory activity against ACHE, BCHE, 15-LOX and α-glucosidase but also highlight the critical need to optimize electronic and structural properties for the development of potent compounds. This detailed analysis sets the foundation for subsequent structure-activity relationship studies, which will guide the design of novel inhibitors with improved therapeutic profiles.

**7h** is characterized by an intermediate level of electronegativity (χ = 0.09686eV) and chemical potential (μ = 0.18548eV), with a substantial absolute hardness (η = 0.08862eV). This hardness suggests a strong resistance to deformation of its electron cloud, making it relatively inflexible in accommodating changes in its electron distribution, as indicated by its low global softness (σ = 11.28410 eV^-1) and moderate electrophilicity index (ω = 0.448 eV). In contrast, **7i** exhibits lower electronegativity (χ = 0.0057eV) and chemical potential (μ = −0.0057eV), paired with the low hardness (η = 0.00254eV) observed indicating a pronounced ability to stabilize additional electron density and thereby enhancing its reactivity under electrophilic attack This pronounced hardness underscores a very robust resistance to electronic reconfiguration, coupled with the lowest global softness (σ = 393.70eV^-1), which correlates with a relatively lower capacity to attract additional electrons, as reflected in its electrophilicity index (ω = 0.0064eV). This detailed electronic structure analysis lays the groundwork for further structure-activity relationship studies, guiding the design of new inhibitors with improved therapeutic profiles.

### 2.7 Dynamic simulations, comprehensive analysis of structural flexibility and stability

**Fig 14:**
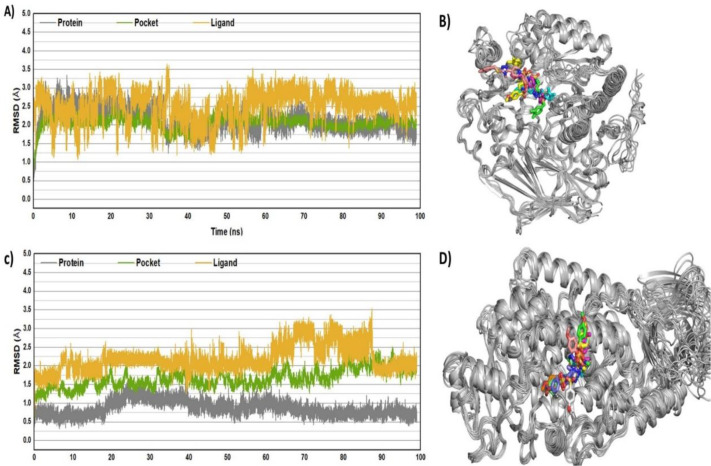
RMSD plots for studied complexes (A) LOX-7h and (B) glucosidase-7h after 100 ns. Post-simulated snapshots from MD trajectories of selected complexes are superposed over co-crystalized structures corresponding complexes (C) LOX-7h and (D) glucosidase-7h.

The results of the RMSD analysis showed that equilibrium was reached in the first five nanoseconds for both systems under investigation, namely 15-LOX-**7h** and α-glucosidase-**7h**. After 30 nanoseconds of simulated molecular dynamics, the systems that involving LOX with **7h** and glucosidase with **7h** compound exhibited comparatively stable fluctuations, suggesting a state of equilibrium. To be more precise, Fig 14A shows that the RMSD values averaged approximately 2.14Å, 1.50Å, and 2.12Å for the protein’s Cα atoms, the binding pocket’s backbone atoms, and the heavy ligands in the LOX-**7h** complex, respectively. On the other hand, the protein’s RMSD values for the α-glucosidase-**7h** complex were approximately ∼1.75Å. Approximately 2.25Å for the ligand and 2.0Å for the binding pocket, as shown in Fig 14C. Based on these findings, it appears that compound **7h** might establish a more robust connection with LOX and α-glucosidase in their individual complexes. The ligand and protein RMSD values both stayed below 3Å, despite some initial variability, and equilibrium was quickly reached. As shown in Figs 14 B and 14D, the stability of these conformations was further verified by superimposing the coordinates of typical MD-simulated snapshots onto their initial conformations. This structural study showed that all complexes and ligands maintained their initial shape and important interactions with neighboring atoms, which were recorded after every 10 ns. These results validate the reliability of MD simulation results for additional binding free energy analysis and offer fascinating insights into the precise processes by which the inhibitors interact with LOX and α-glucosidase.

**Fig 15:**
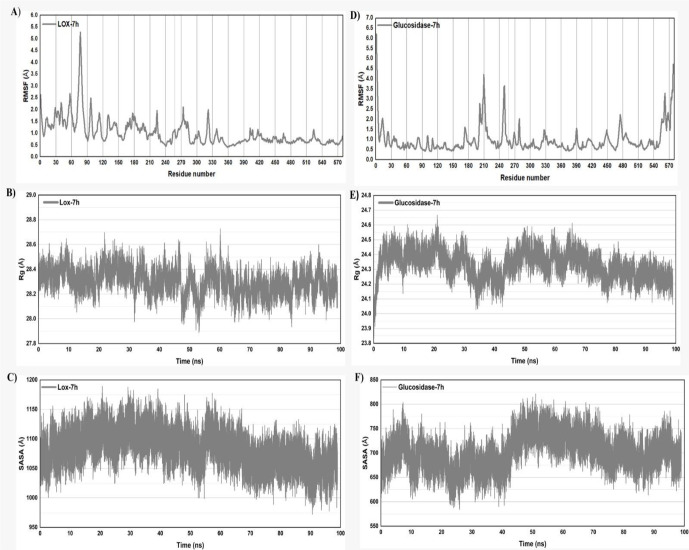
(A and B) Root mean fluctuation (RMSF) curve for selected complexes LOX-7h and glucosidase-7h. (C and D),(E and F) Plots for radius of gyration (Rg) and solvent accessible surface area (SASA) for both complexes respectively.

Both the LOX-**7h** complex system and α-glucosidase-**7h** complex system simulations revealed their own specific fluctuation patterns across the residues, indicating that **7h** compound may interact by different affinity ways with the protein’s structural dynamics. There are notable peaks in flexibility around the same residues for both ligands, although the amplitudes of these peaks vary. Specifically, peaks around residues 40–80, 100–220, and 240–330 show significant fluctuations for LOX-**7h** complex as illustrated in Fig 15, and the fluctuations in the α-glucosidase-**7h** complex are consistently higher, particularly around residues 210 and 250 as illustrated in Fig 15D. Between these peaks, the RMSF values are relatively low for α-glucosidase-**7h** complex, which implies that these regions of the protein remain stable and rigid during the simulations. The variations in RMSF could indicate differences in binding affinities or stabilities within the enzyme-inhibitor complex. Higher fluctuations in both complexes might imply a loose binding that allows more movement, or that **7h** binding influences more regions of the enzyme. Such changes in the flexibility of specific residues can affect the enzyme’s functionality, potentially altering how efficiently it catalyzes its reactions. This analysis revealed that the dynamic characteristics and RMSF distributions across the protein structures of **7h** compound systems follow their specific patterns according to their binding modes with both enzymes. Specifically, the domain lies within residues 80–220 is crucial for protein stability and folding in LOX enzyme, while the domain lies within residues 210–250 is crucial for protein stability and folding in α-glucosidase enzyme. These domain aids in ligand interactions, and its dynamics during simulations reveal how it affects overall protein function. Molecular dynamics simulations of these domains are key to understanding the dynamic nature of protein functions, crucial for drug design and biological research.

To investigate how ligand binding affects the protein’s structural compactness, Figs 15B and 15F display the graph of the radius of gyration (Rg) over time for the LOX-**7h** and α-glucosidase-**7h** complex over a simulation period of 100 nanoseconds (ns). The average Rg values for the two complexes were approximately ∼28.4–28.7Å and ∼24.0–24.7Å, respectively. The lower values for the α-glucosidase-**7h** complex indicate a more compact structure and outstanding regularity. Throughout the simulation, the Rg values for both complexes stayed aligned in a distinct pattern, suggesting that **7h** has a distinct and non-significant impact on the protein’s overall structure and compactness.

Additionally, a temporal analysis of the solvent-accessible surface area (SASA) was conducted to gain a deeper comprehension of the interactions between the LOX-**7h** and α-glucosidase-**7h** complex under examination (Fig 15C and 15F). The SASA value of the LOX bound complex is approximately ∼1050–1150̅, suggesting a stable contact and a slightly elevated solvent exposure of the protein surface. The SASA value of the α-glucosidase bound complex is approximately ∼580–800Å, suggesting a relatively mild or less variable solvent exposure of the protein surface. While the α-glucosidase complex has a lower total SASA, suggesting a more compact conformation or less exposure to the solvent, potentially indicating tighter binding or less structural flexibility, this could indicate diverse conformational states or a more dynamic interaction with the solvent. The (Figs 15A-15B, 15A–15F) detailing RMSD, RMSF, radius of gyration, and SASA provide insights into the dynamics and structural properties of LOX and α-glucosidase in interaction with **7h** during molecular dynamics simulations. These analyses are essential for understanding the protein’s function, stability, and how it binds drugs. Together, these metrics illustrate a detailed narrative of the protein’s behavior in solution, particularly its response to ligands. Through a analysis of the dynamic and structural influences of compound **7h** with both enzymes, we can identify crucial structural elements necessary for effective inhibition of LOX and α-glucosidase. This understanding is foundational for developing potent inhibitors with significant therapeutic value.

### 2.8 Binding free energy analysis

**Fig 16:**
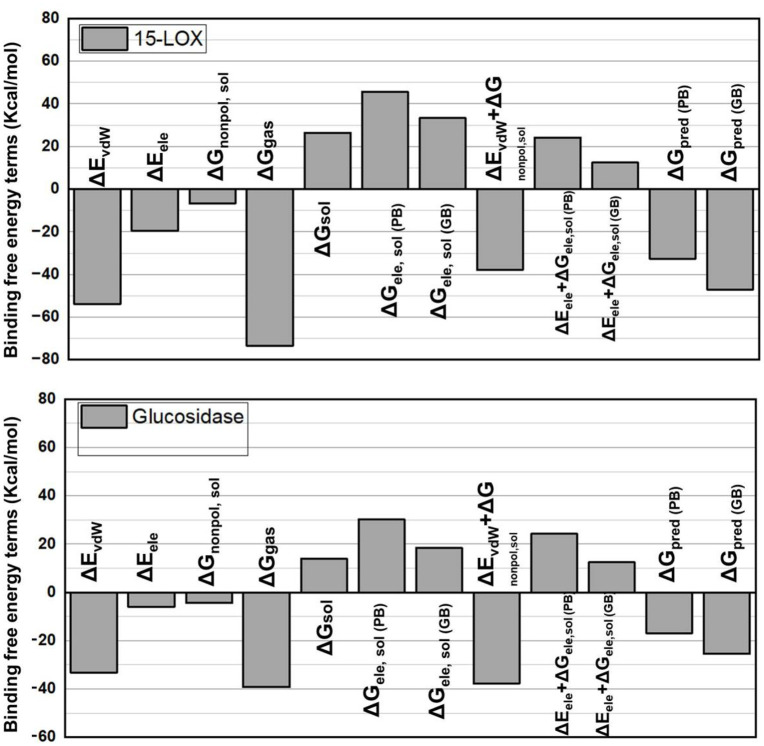
Binding free energy terms complexes LOX-7h and glucosidase-7h.

The binding free energy components for two complexes, LOX-**7h** and α-glucosidas-**7h**, are plotted on the graph in Fig 16. The units of measurement for the energy terms are kilocalories per mole (kcal/mol). Among them are Van der Waals energy, or ΔEvdw, is a component that shows the energy of non-covalent interactions between ligands and proteins. Greater interactions are suggested by lower numbers. Higher negative values for LOX and α-glucosidase, respectively, show stronger Van der Waals interactions with compound **7h**. These values indicate that LOX has stronger Van der Waals interactions with compound **7h** and consequently a more efficient or close packing of both proteins. Electrostatic energy, or ΔE_ele, is a measure of the electrostatic interactions between the ligand and the charged groups in proteins. Once more, higher negative values indicate more robust interactions. For example, **7h** with LOX (−19.6255 kcal/mol) exhibits a significantly higher electrostatic interaction compared to α-glucosidase (−5.9691 kcal/mol), indicating superior charge complementarity with the protein. ΔG_gas, which is the sum of ΔE_vdw and ΔE_ele, is the total gas-phase free energy and it indicates the change in free energy caused by the binding process in the gas phase. When compared to α-glucosidase-**7h**, which has a negative value of about 40 kcal/mol, LOX-**7h** has a higher value of −56.5586 kcal/mol, indicating a better binding affinity without solvent effects in the gas phase. When a complex transitions from a gas phase to a solvated state, its ΔG_sol represents the solvation free energy. The total binding free energy is represented by ΔG_total, which is the sum of ΔG_gas and ΔG_sol. ΔE_MM/GBSA (for polar solvation, ΔE_MM/PBSA). For polar contributions, ΔG_polar and ΔG_nonpolar, molecular mechanics energies are combined with generalized Born and surface area continuum solvation (GBSA) or Poisson-Boltzmann surface area (PBSA): The solvation energy inside GBSA/PBSA, ΔE_ele + ΔG_polar (PB/GB), has both polar and nonpolar components. The electrostatic contribution to solvation can be understood by adding the polar solvation energy and electrostatic energy. This represents the solution of energy’s electrostatic component.

In the Poisson-Boltzmann (PB) and Generalized Born (GB) models, atorvastatin exhibits marginally better values, indicating that solvation has less of an adverse effect on it. The impact of different molecular interactions on overall binding affinity is demonstrated by the analysis of binding energies for 7 hours with LOX and α-glucosidase. Even though **7 h**’s van der Waals and nonpolar solvation energies indicate stronger individual interactions, its overall binding free energy is more favorable when compared to **7 h**’s energy with LOX. This implies that **7h** interacts with the enzyme in a more balanced manner, thereby incorporating important solvation dynamics that are critical in physiological settings. This assessment emphasizes the value of a thorough approach to drug design, with an emphasis on ligand optimization to improve therapeutic efficacy and specificity against α-glucosidase and LOX. **7h** is a strong contender for therapeutic applications targeting anti-inflammatory and anti-asthmatic medications because of its capacity to form a stable and efficient complex with the enzymes. These findings demonstrate that while van der Waals and hydrophobic interactions are more significant in determining the inhibitory potential of LOX and α-glucosidase, electrostatic interactions still play a role in the binding process. This work provides insights into the binding mechanisms and, by emphasizing the reinforcement of van der Waals and hydrophobic interactions, suggests targeted strategies for the synthesis of more potent LOX and α-glucosidase inhibitors.

### 2.9 Protein binding studies

Triazole based compounds have vast biological impacts that include inhibiting platelet aggregation and proliferation, promoting cancer cell differentiation and death, and having antibacterial, antispasmodic, anti-inflammatory, and anti-inflammatory capabilities [41–43]. The interaction of many triazole derivatives was investigated in the current work. A BSA binding research was conducted to offer some useful insights into the pharmacological and pharmacodynamic therapeutic effects of these drugs. A few triazole derivatives’ binding tests with BSA were evaluated to understand that how various substituents attached to ring of triazole impact interactions between them and the globular protein. Using fluorometric BSA titrations with different derivative concentrations (0–5.57 × 10−4 M), the interaction of the derivatives of triazole with BSA was determined. Various mechanisms can lead to either static or dynamic quenching of fluorescence. These include ground state complex formation, molecular reorganization, excited state reactions, and transfer of energy. The Stern-Volmer equation (1) is typically used to interpret the kind of quenching process [44,45].


$$\frac{{\text{F}}_{\text{0}}}{\text{F}}\;=\text{1}+{\text{K}}_{\text{SV}}[\text{Q}]={\text{K}}_{\text{q }}\tau_{\text{o}}[\text{Q}]+\;\text{1}$$ (1)

Where τo is the mean survival time of the biological molecules without the quencher, which has a range of 10–8 s, and [Q] is quencher concentration, Kq is observed bimolecular quenching’s rate constant, and F and Fo are the BSA fluorescence intensities after and before addition of quencher, correspondingly. The number of sites for binding in produced compounds (n) and the constant of binding (Ka) may be computed using the second-order logarithm formula (2).


$$\text{Log }[({\text{F}}_{\text{0}}–\text{F})/\text{ F}]=\text{log }{\text{K}}_{\text{a}}+\text{n log}[\text{Q}]$$ (2)

**Fig 17:**
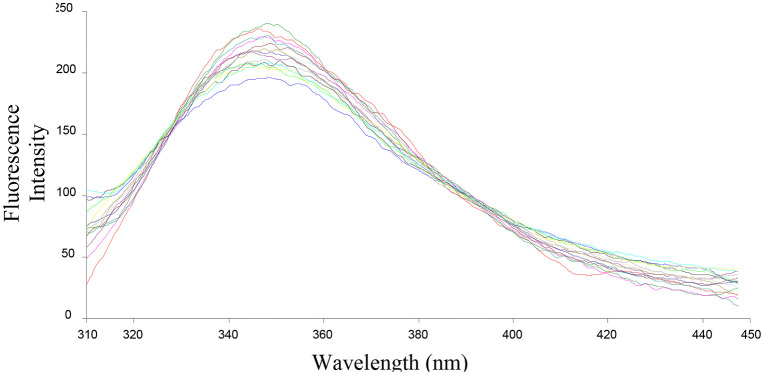
Fluorescence spectra of BSA-7b complex at varying wavelengths.

**Fig 18:**
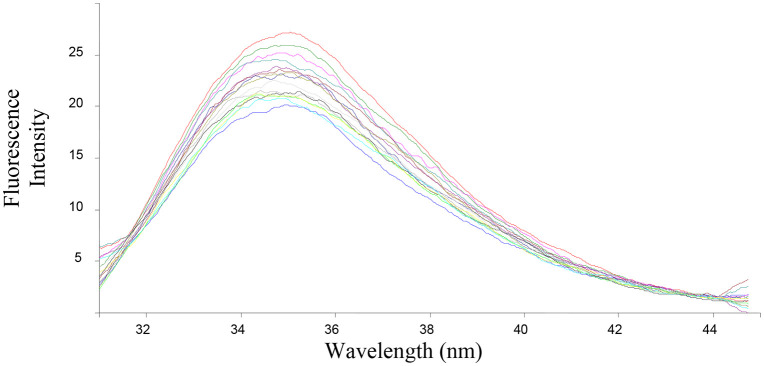
Fluorescence spectra of BSA-7e complex at varying wavelengths.

**Fig 19:**
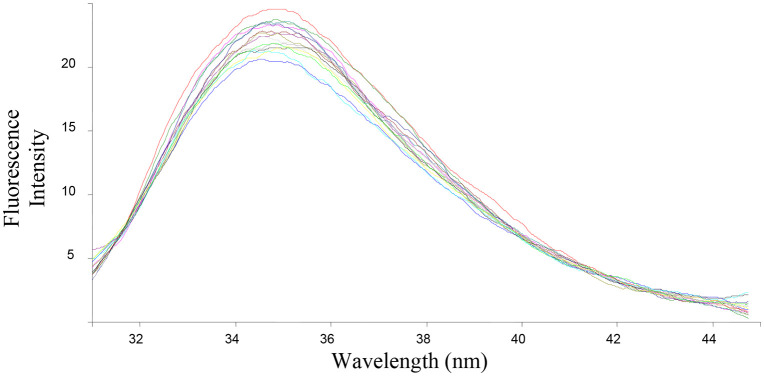
Fluorescence spectra of BSA-7f complex at varying wavelengths.

**Fig 20:**
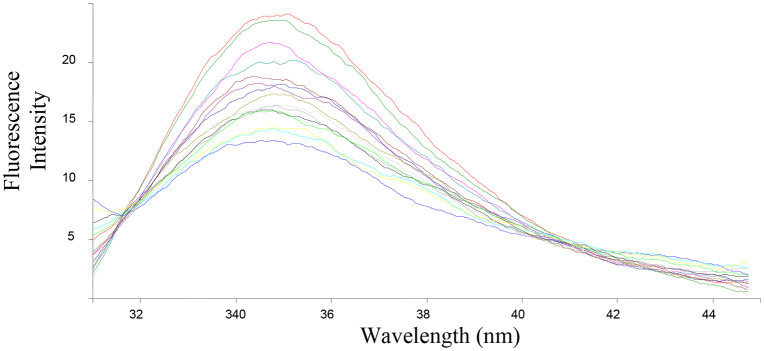
Fluorescence spectra of BSA-7g complex at varying wavelengths.

**Fig 21:**
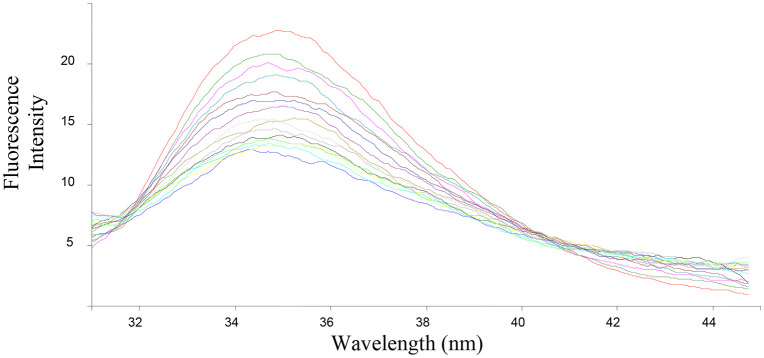
Fluorescence spectra of BSA-7j complex at varying wavelengths.

**Fig 22:**
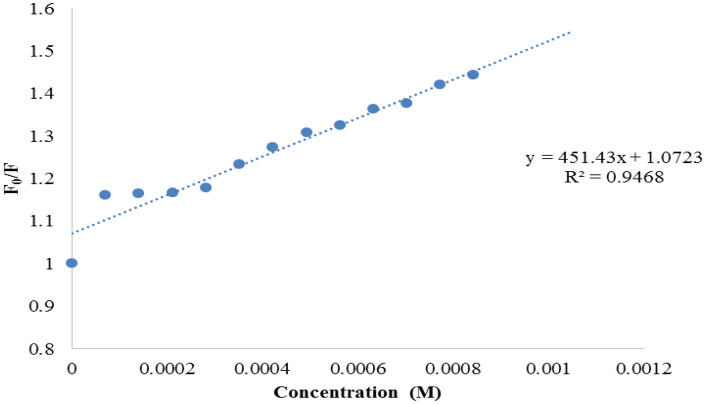
Plots of Stern-Volmer for compound 7b.

**Fig 23:**
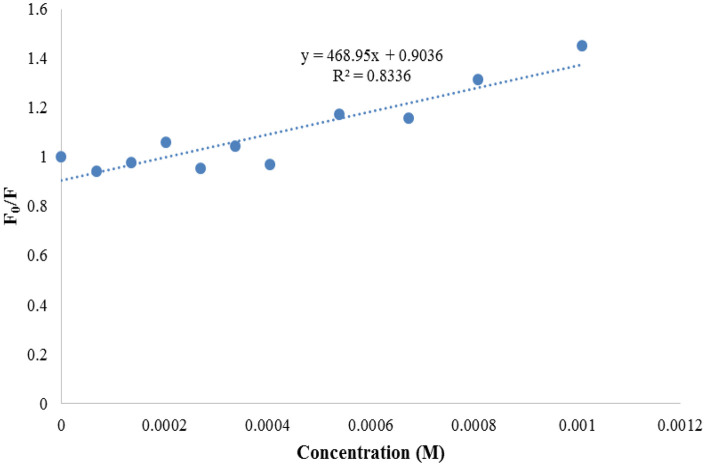
Plots of Stern-Volmer for compound 7e.

**Fig 24:**
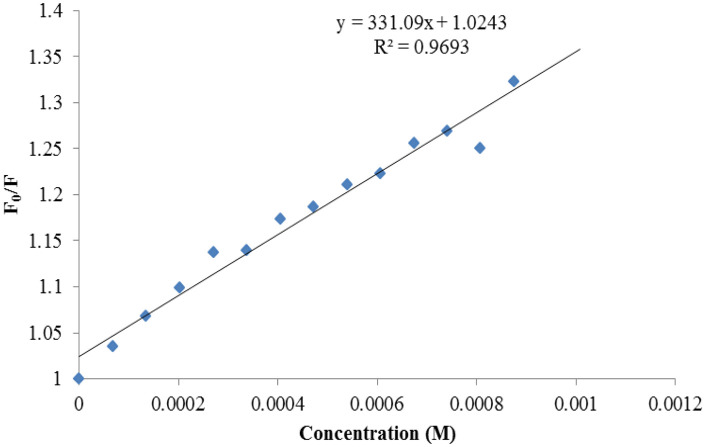
Plots of Stern-Volmer for compound 7f.

**Fig 25:**
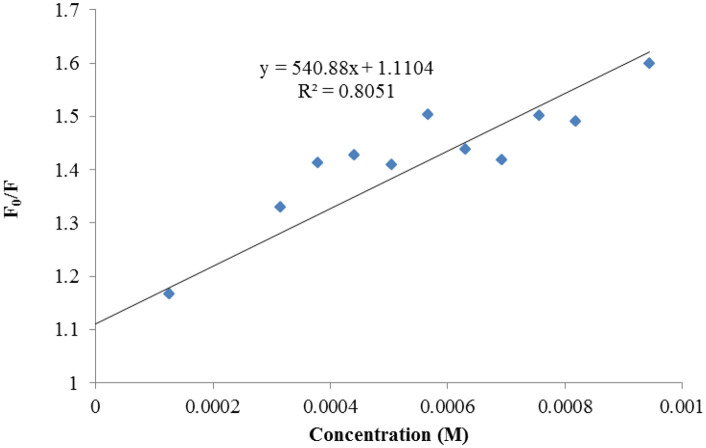
Plots of Stern-Volmer for compound 7g.

**Fig 26:**
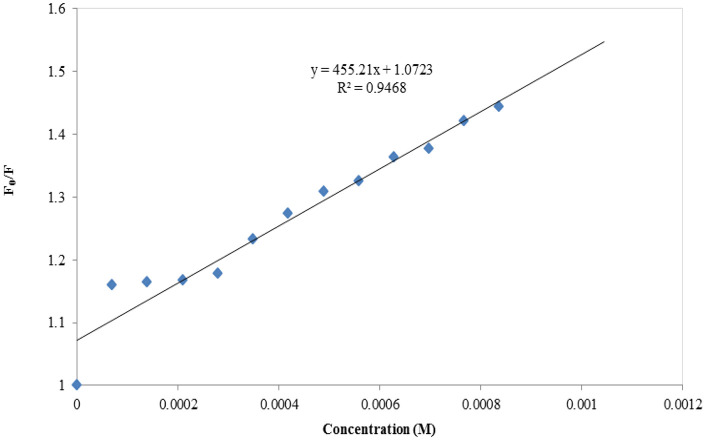
Plots of Stern-Volmer for compound 7j.

The ability of the compounds based on 1,2,4-triazoles to interact with BSA was shown to be affected by the various triazole ring substituents. Among the series of ten compounds, five molecules were tested for their BSA binding potential (Table 6, Fig 17, 18, 19, 20, 21, 22, 23, 24, 25, 26). Compound **7e** revealed the highest binding affinity with the BSA. This might be because compound **7e** is able to bind to two binding sites on BSA molecule (n = 2) compared to other derivatives.

**Table 6:** Number of binding sites, Stern-Volmer quenching constants, and binding constant for the chosen compounds (7b, 7e, 7f, 7g, 7j).

Compounds	K_SV _× 10^1^ (M^-1^)	k_q_ × 10^10^(M^-1^ s^-1^)	K_a_(M^-1^)	N
7b	4.51	4.51	2.78 × 10^1^	0.58
7e	4.69	4.69	2.44 × 10^6^	2.24
7f	3.31	3.31	9.77 × 10^1^	0.82
7g	5.41	5.41	2.91 × 10^1^	0.56
7j	4.55	4.55	3.63 × 10^1^	0.63

## 3 Experimental

### 3.1 General information

Unless otherwise noted, the analytical grade research chemicals including solvents and reagents were availed through the supplier of local market. Silica gel coated aluminum plates were utilized to evaluate reaction progress. The structural conformations of designed compounds were made using ^1^H-NMR along with the assistance of ^13^C-NMR to elaborate the structures recorded through Bruker AM-400 spectrometer in CDCl_3_. The chemical shift measured in parts per million and included several peaks and signals, provided justification for the NMR results. The availability of different functionalities in the synthesized library of compounds was justified by the help of IR spectroscopy through Jasco-320-A spectrophotometer. Varioskan Flash 4.00.53 assisted with fluorescence studies of the compounds presented herein.

### 3.2 Synthesis

#### 3.2.1 Synthesis of substituted carboxylate (3).

The stirring of compound **1** (0.06 mol, 4-methoxybenzenesulfonyl chloride) and compound **2** (0.06 mol, ethyl isonipecotate) in 10% Na_2_CO_3_ aqueous solution at room temperature yielded a good amount of title compound, **3**. TLC was used to validate the purity of compounds that were synthesized during experimentation. Following reaction completion and the resultant mixture neutralization with diluted HCl, targeted chemicals in the form of white precipitates were generated. After filtering and washing with cold distilled water, the precipitates were placed to dry at room temperature [46].

#### 3.2.2 Synthesis of substituted-carbohydrazide (4).

Compound **4** was availed through the refluxed reaction of compound **3** (0.04 mol) with hydrazine hydrate for five hours using methanol as the solvent. TLC confirmed the speed and status the course of the reaction. To create the compound **4** precipitates, large amount of cold water was incorporated after the surplus solvent was removed by distillation, which was then stored and used for further reactions.

#### 3.2.3 Synthesis of 2,5-disubstituted-1,2,4-triazole (5).

The target compounds **7a-j** was obtained by utilizing the equimolar (0.03 mol) compound **4** and methyl isothiocyanate in methanol to achieve the title compound **5**, through a reflux reaction which was monitored using thin layer chromatography. Finally, dil. HCl was added to finish the reaction with stirring continuously, resulting in a pH of 4–5. After being obtained as precipitates, the product was filtered and allowed to dry at ambient temperature

#### 3.2.4 Synthesis of library of 1,2,4-triazole hybrids (7a-j).

**Conventional method.** Aimed compounds were synthesized by agitating equimolar compound **5** (0.6 mmol, activated by LiH) with aralkyl halides (**6a-j**) in DMF for 420–960 minutes at room temperature. TLC tracked the reaction’s progress. Depending on the type of the products, the intended products, **7a–j**, were obtained as precipitates or extracts when the reaction was completed. The resultant products were set up for biological research and structural elucidation.

**Microwave method.** Aimed compounds **7a–j** were synthesized by stirring equimolar compound **5** (0.6 mmol, activated by LiH) with aralkyl halides **6a–j** in DMF for 56–73 seconds at room temperature. TLC monitored the reaction’s progress. Depending on the nature of the products that were saved for structural elucidation and biological research, the targeted compounds were acquired as precipitates. In comparison to conventional synthesis, the compounds created in a relatively brief period with the help of microwave assisted synthesis showed exceptional yields and purity.

***3-Benzylthio-5-{1-[(4-methoxyphenyl)sulfonyl]-4-piperidinyl}-4-methyl-4H-1,2,4-triazole (7a).*** Off white amorphous solid; yield: 91%; M.P: 142^o^C; HRMS: [M]^•+^ 458.1439 (Calcd. for C_22_H_26_N_4_O_3_S_2_; 458.1446); Anal. Calcd. for C_22_H_26_N_4_O_3_S_2_: C, 57.37; H, 6.13; N, 12.16; O, 10.42; S, 13.92; found C, 57.33; H, 6.09; N, 12.14; O, 10.39; S, 13.88; IR (KBr, υ_*max*_, cm^-1^): 2806 (Aromatic carbon hydrogen single bond), 1605 (Carbon nitrogen double bond), 1524 (Aromatic carbon carbon double bond), 1362 (Methyl group), 1315 (Sulphur monoxide), 1204 (Carbon oxygen single bond); ^1^H-NMR (CDCl_3_, 600 MHz, δ/ppm) (coupling constant, *J* = Hertz): ^1^H-NMR *δ* (ppm)): 7.69 (d, coupling constant = 8.1 Hertz, 2H, 2’, 6’), 7.26-7.25 (m, 3H, 3’‘, 4’‘, 5’‘), 7.21 (d, coupling constant = 6.9 Hz, 2H, 2’‘, 6’‘), 7.18 (d, coupling constant = 8.2 Hz, 2H, 3’, 5’), 4.23 (s, 2H, 3’‘‘), 3.86 (s, 3H, 1’‘‘), 3.62-3.60 (m, 2H, 2e, 6e), 3.81 (s, 3H, 2’‘‘), 2.79-2.76 (m, 1H, 4), 2.41-2.37 (m, 2H, 2a, 6a), 1.88-1.86 (m, 2H, 3e, 5e), 1.72-1.67 (m, 2H, 3a, 5a); ^13^C-NMR (CDCl_3,_ 150 MHz, δ/ppm): 162.68 (4’), 157.80 (5’‘‘‘), 148.44 (3’‘‘‘), 137.22 (1’‘), 129.70 (2’, 6’), 128.81 (2’‘, 6’‘), 128.38 (3’‘, 5’‘), 127.39 (4’‘), 126.79 (1’), 114.49 (3’, 5’), 55.69 (1’‘‘), 45.43 (2, 6), 37.54 (3’‘‘), 30.48 (4), 29.51 (2’‘‘), 28.82 (3, 5).

***3-[(2-Methylbenzyl)thio]-5-{1-[(4-mthoxyphenyl)soulfonyl]-4-piperidinyl}-4-methyl-4H-1,2,4-triazole (7b)*.** Off white amorphous product; yield: 89%; M.P: 220.1^o^C; HRMS: [M]^•+^ 472.1592 (Calcd. forC_23_H_28_N_4_O_3_S_2_; 472.1603); Anal. Calcd. for C_23_H_28_N_4_O_3_S_2_: C, 58.45; H, 5.97; N, 11.85; O, 10.16; S, 13.57; found C, 58.45; H, 5.92; N, 11.80; O, 10.11; S, 13.51; IR (KBr, υ_*max*_, cm^-1^): 2810 (Aromatic carbon hydrogen single bond), 1610 (Carbon nitrogen double bond), 1530 (Aromatic carbon double bond), 1350 (Methyl group), 1300 (Sulphur monoxide), 1200 (Carbon oxygen single bond); ^1^H-NMR (CDCl_3,_ 600 MHz, δ/ppm) (coupling constant, *J* = Hertz): 7.70 (d, coupling constant = 7.9 Hz, 2H, 2’, 6’), 7.18 (d, coupling constant = 8.1 Hz, 2H, 3’, 5’), 7.05–6.95 (m, 4H, 3’‘ to 6’‘), 4.24 (s, 1H, 3’“), 3.86 (s, 3H, 1’“), 3.62–3.60 (m, 2H, 2 _e_, 6_e_), 3.25 (s, 3H, 2’“), 2.79–2.79 (m, 1H, 4), 2.41–2.37 (m, 2H, 2a, 6a), 2.24 (s, 3H, 4’“), 1.89–1.87 (m, 2H, 3e, 5e), 1.73–1.67 (m, 2H, 3a, 5a); ^13^C-NMR (CDCl_3,_ 150 MHz, δ/ppm): 162.58 (4’), 157.88 (5’“‘), 148.09 (3’“‘), 136.49 (1’‘), 132.01 (2’‘), 131.05 (3’‘), 130.86 (4’‘), 130.27 (6’‘), 129.70 (2’, 6’), 128.30 (5’‘), 126.49 (1’), 114.49 (3’, 5’), 55.69 (1’“), 45.43 (2, C-6), 36.40 (3’“), 30.47 (4), 29.61 (2’“), 28.82 (3, 5).

***3-[(3-Methylbenzyl)thio]-5-{1-[(4-methoxyphenyl)sulfonyl]-4-piperidinyl}-4-methyl-4H-1,2,4-triazole (7c).*** Off white amorphous solid; yield: 88%; M.P: 135.0^o^C; HRMS: [M]^•+^ 472.1592 (Calcd. for C_23_H_28_N_4_O_3_S_2_; 472.1603); Anal. Calcd. for C_23_H_28_N_4_O_3_S_2_: C, 58.45; H, 5.97; N, 11.85; O, 10.16; S, 13.57; found C, 58.45; H, 5.92; N, 11.80; O, 10.11; S, 13.51; IR (KBr, υ_*max*_, cm^-1^): 2810 (Aromatic carbon hydrogen single bond), 1610 (Carbon nitrogen double bond), 1520 (Aromatic carbon double bond), 1360 (Methyl group), 1310 (Sulphur monoxide), 1200 (Carbon oxygen single bond); ^1^H-NMR (CDCl_3,_ 600 MHz, δ/ppm) (coupling constant, *J* = Hertz): 7.70 (d, coupling constant = 7.5 Hz, 2H, 2’, 6’), 7.18–7.14 (m, 3H, 3’, 5’, 5’‘), 7.06 (d, coupling constant = 7.4 Hz, 1H, 6’‘), 7.01-6.99 (m, 2H, 2’‘, 4’‘), 4.18 (s, 1H, 3’“), 3.86 (s, 3H, 1’“), 3.62–3.60 (m, 2H, 2e, 6e), 3.20 (s, 3H, 2’“), 2.80–2.78 (m, 1H, 4), 2.41–2.37 (m, 2H, 2a, 6a), 2.23 (s, 3H, 4’“), 1.89–1.87 (m, 2H, 3e, 5e), 1.73–1.69 (m, 2H, 3a, 5a); ^13^C-NMR (CDCl_3,_ 150 MHz, δ/ppm): 162.68 (4’), 157.77 (5’“‘), 148.50 (3’“‘), 137.53 (1’‘), 137.02 (3’‘), 129.70 (2’, 6’), 129.37 (5’‘), 128.30 (6’‘), 128.01 (4’‘), 126.80 (1’), 125.90 (2’‘), 114.49 (3’, 5’), 55.69 (1’“), 45.43 (2,6), 37.60 (3’“), 30.51 (4), 29.52 (2’“), 28.83 (3,5), 20.84 (4’“).

***3-[(4-Methylbenzyl)thio]-5-{1-[(4-methoxyphenyl)sulfonyl]-4-piperidinyl}-4-methyl-4H-1,2,4-triazole (7d).*** White amorphous solid; yield: 85%; M.P: 146.0^o^C; HRMS: [M]^•+^ 472.1592 (Calcd. for C_23_H_28_N_4_O_3_S_2_; 472.1603); Anal. Calcd. for C_23_H_28_N_4_O_3_S_2_: C, 58.45; H, 5.97; N, 11.85; O, 10.16; S, 13.57; found C, 58.45; H, 5.92; N, 11.80; O, 10.11; S, 13.51; IR (KBr, υ_*max*_, cm^-1^): 2800 (Aromatic carbon hydrogen single bond), 1600 (Carbon nitrogen double bond), 1520 (Aromatic carbon double bond), 1360 (Methyl group), 1310 (Sulphur monoxide), 1210 (Carbon oxygen single bond); ^1^H-NMR (CDCl_3,_ 600 MHz, δ/ppm) (coupling constant, *J* = Hertz): 7.70 (d, coupling constant = 8.2 Hz, 2H, 2’, 6’), 7.17 (d, coupling constant = 8.3 Hz, 2H, 3’, 5’), 7.10–7.06 (m, 4H, 2’‘, 3’‘, 5’‘, 6’‘), 4.19 (s, 1H, 3’“), 3.86 (s, 3H, 1’“), 3.62–3.60 (m, 2H, 2e, 6e), 3.20 (s, 3H, 2’“), 2.79–2.77 (m, 1H, 4), 2.41–2.25 (m, 2H, 2a, 6a), 2.25 (s, 3H, 4’“), 1.89–1.86 (m, 2H, 3e, 5e), 1.71–1.69 (m, 2H, 3a, 5a); ^13^C-NMR (CDCl_3,_ 150 MHz, δ/ppm): 162.68 (4’), 157.75 (5’“‘), 148.53 (3’“‘), 136.64 (1’‘), 134.05 (4’‘), 129.70 (2’, 6’), 128.92 (2’‘, 6’‘), 128.73 (3’‘, 5’‘), 126.80 (1’) 114.49 (3’, 5’), 55.69 (1’“), 45.43 (2,6), 37.29 (3’“), 30.48 (4), 29.54 (2’“), 28.83 (3,5), 20.65 (4’“).

***3-[(2-Chlorobenzyl)thio]-5-{1-[(4-methoxyphenyl)sulfonyl]-4-piperidinyl}-4-methyl-4H-1,2,4-triazole (7e).*** White amorphous solid; yield: 87%; MP: 66.0^o^C; HRMS: [M]^•+^ 492.1051 (Calcd. for C_22_H_25_ClN_4_O_3_S_2_; 492.1057); Anal. Calcd. for C_22_H_25_ClN_4_O_3_S_2_: C, 53.59; H, 5.11; Cl, 7.19; N, 11.36; O, 9.74; S, 13.01; found C, 53.54; H, 5.09; Cl, 7.16; N, 11.32; O, 9.71; S, 12.08; IR (KBr, υ_*max*_, cm^-1^): 2800 (Aromatic carbon hydrogen single bond), 1610 (Carbon nitrogen double bond), 1530 (Aromatic carbon double bond), 1360 (Methyl group), 1300 (Sulphur monoxide), 1210 (Carbon oxygen single bond), 705 (Carbon chloride); ^1^H-NMR (CDCl_3,_ 600 MHz, δ/ppm) (coupling constant, *J* = Hertz): 7.70 (d, coupling constant = 7.9 Hz, 2H, 2’, 6’), 7.44 (d, coupling constant = 7.8 Hz, 1H, 3’‘), 7.30-7.22 (m, 3H, 4’‘, 5’‘, 6’‘), 7.18 (d, coupling constant = 8.1 Hz, 2H, 3’, 5’), 4.29 (s, 1H, 3’“), 3.86 (s, 3H, 1’“), 3.62–3.60 (m, 2H, 2e, 6e), 3.20 (s, 3H, 2’“), 2.79–2.78 (m, 1H, 4), 2.41–2.38 (m, 2H, 2a, 6a), 1.88–1.86 (m, 2H, 3e, 5e), 1.73–1.69 (m, 2H, 3a, 5a); ^13^C-NMR (CDCl_3,_ 150 MHz, δ/ppm): 162.68 (4’), 158.02 (5’“‘), 147.86 (3’“‘), 134.64 (1’‘), 133.03 (2’‘), 131.24 (3’‘), 129.70 (2’, 6’), 129.53 (4’‘), 129.41 (5’‘), 127.26 (6’‘), 126.79 (1’), 114.49 (3’, 5’), 55.70 (1’“), 45.42 (2,6), 35.62 (3’“), 30.51 (4), 29.51 (2’“), 28.80 (3, 5).

***3-[(4-Chlorobenzyl)thio]-5-{1-[(4-methoxyphenyl)sulfonyl]-4-piperidinyl}-4-methyl-4H-1,2,4-triazole (7f).*** White amorphous solid; yield: 89%; M.P: 233.2^o^C; HRMS: [M]^•+^ 492.1051 (Calcd. for C_22_H_25_ClN_4_O_3_S_2_; 492.1057); Anal. Calcd. for C_22_H_25_ClN_4_O_3_S_2_: C, 53.59; H, 5.11; Cl, 7.19; N, 11.36; O, 9.74; S, 13.01; found C, 53.54; H, 5.09; Cl, 7.16; N, 11.32; O, 9.71; S, 12.08; IR (KBr, υ_*max*_, cm^-1^): 2800 (Aromatic carbon hydrogen single bond), 1600 (Carbon nitrogen double bond), 1510 (Aromatic carbon double bond), 1350 (Methyl group), 1300 (Sulphur monoxide), 1200 (Carbon oxygen single bond), 697 (Carbon chloride); ^1^H-NMR (CDCl_3,_ 600 MHz, δ/ppm) (coupling constant, *J* = Hertz): 7.70 (d, coupling constant = 7.4 Hz, 2H, 2’, 6’), 7.33 (d, coupling constant = 7.4 Hz, 2H, 3’‘, 5’‘), 7.26 (d, coupling constant = 7.7 Hz, 2H, 2’‘, 6’‘), 7.18 (d, coupling constant = 7.7 Hz, 2H, 3’, 5’), 4.24 (s, 1H, 3’“), 3.86 (s, 3H, 1’“), 3.62–3.60 (m, 2H, 2e, 6e), 3.25 (s, 3H, 2’“), 2.79–2.77 (m, 1H, 4), 2.41–2.37 (m, 2H, 2a, 6a), 1.89–1.87 (m, 2H, 3e, 5e), 1.72–1.66 (m, 2H, 3a, 5a); ^13^C-NMR (CDCl_3,_ 150 MHz, δ/ppm): 162.68 (4’), 160.11 (5’“‘), 158.01 (3’“‘), 134.49 (1’‘), 134.55 (4’‘), 130.70 (3’‘, 5’‘), 129.70 (2’, 6’), 128.30 (2’‘, 6’‘), 126.70 (1’), 114.49 (3’, 5’), 55.69 (1’“), 45.43 (2,6), 36.40 (3’“), 30.47 (4), 29.61 (2’“), 28.81 (3,5).

***3-[(2,4-Dichlorobenzyl)thio]-5-{1-[(4-methoxyphenyl)sulfonyl]-4-piperidinyl}-4-methyl-4H-1,2,4-triazole (7g).*** White amorphous solid; yield: 90%; M.P: 186.2^o^C; HRMS: [M]^•+^ 526.0659 (Calcd. for C_22_H_24_Cl_2_N_4_O_3_S_2_; 526.0667); Anal. Calcd. for C_22_H_24_Cl_2_N_4_O_3_S_2_: C, 50.09; H, 4.59; Cl, 13.44; N, 10.62; O, 9.10; S, 12.16; found C, 50.05; H, 4.55; Cl, 13.40; N, 10.60; O, 9.05; S, 12.12; IR (KBr, υ_*max*_, cm^-1^): 2800 (Aromatic carbon hydrogen single bond), 1620 (Carbon nitrogen double bond), 1500 (Aromatic carbon double bond), 1340 (Methyl group), 1310 (Sulphur monoxide), 1200 (Carbon oxygen single bond), 700 (Carbon chloride); ^1^H-NMR (CDCl_3,_ 600 MHz, δ/ppm) (coupling constant, *J* = Hertz): 7.70 (d, coupling constant = 8.8 Hz, 2H, 2’, 6’), 7.61 (d, coupling constant = 2.0 Hz, 1H, 3’‘), 7.33 (dd, coupling constant = 8.2, 2.1 Hz, 1H, 5’‘), 7.29 (d, coupling constant = 8.2 Hz, 1H, 6’‘), 7.18 (d, coupling constant = 8.8 Hz, 2H, 3’, 5’), 4.28 (s, 1H, 3’“), 3.86 (s, 3H, 1’“), 3.62–3.60 (m, 2H, 2e, 6e), 3.27 (s, 3H, 2’“), 2.82–2.79 (m, 1H, 4), 2.42–2.38 (m, 2H, 2a, 6a), 1.89–1.87 (m, 2H, 3e, 5e), 1.73–1.67 (m, 2H, 3a, 5a); ^13^C-NMR (CDCl_3,_ 150 MHz, δ/ppm): 162.68 (4’), 158.10 (5’“‘), 147.63 (3’“‘), 137.66 (1’‘), 133.98 (4’‘), 133.07 (2’‘), 132.51 (3’‘), 129.70 (2’, 6’), 128.84 (5’‘), 127.39 (6’‘), 126.79 (1’), 114.49 (3’, 5’), 55.70 (1’“), 45.42 (2,6), 34.78 (3’“), 30.51 (4), 29.62 (2’“), 28.80 (3, 5).

***3-[(3,4-Dichlorobenzyl)thio]-5-{1-[(4-methoxyphenyl)sulfonyl]-4-piperidinyl}-4-methyl-4H-1,2,4-triazole (7h).*** Solid; yield: 90%; M.P: 178.0^o^C; HRMS: [M]^•+^ 526.0659 (Calcd. for C_22_H_24_Cl_2_N_4_O_3_S_2_; 526.0667); Anal. Calcd. for C_22_H_24_Cl_2_N_4_O_3_S_2_: C, 50.09; H, 4.59; Cl, 13.44; N, 10.62; O, 9.10; S, 12.16; found C, 50.05; H, 4.55; Cl, 13.40; N, 10.60; O, 9.05; S, 12.12; IR (KBr, υ_*max*_, cm^-1^): 2810 (Aromatic carbon hydrogen single bond), 1620 (Carbon nitrogen double bond), 1510 (Aromatic carbon carbon double bond), 1330 (Methyl group), 1300 (Sulphur monoxide), 1200 (Carbon oxygen single bond), 710 (Carbon chloride); ^1^H-NMR (CDCl_3,_ 600 MHz, δ/ppm) (coupling constant, *J* = Hertz): 7.69 (d, coupling constant = 8.8 Hz, 2H, 2’, 6’), 7.54 (d, coupling constant = 8.2 Hz, 1H, 5’‘), 7.50 (d, coupling constant = 1.98 Hz, 1H, 2’‘), 7.26 (dd, coupling constant = 8.2, 2.0 Hz, 1H, 6’‘), 7.17 (d, coupling constant = 8.9 Hz, 2H, 3’, 5’), 4.23 (s, 2H, 3’“), 3.86 (s, 3H, 1’“), 3.62–3.60 (m, 2H, 2e, 6e), 3.29 (s, 3H, 2’“), 2.81–2.79 (m, 1H, 4), 2.42–2.38 (m, 2H, 2a, 6a), 1.90–1.87 (m, 2H, 3e, 5e), 1.72–1.69 (m, 2H, 3a, 5a); ^13^C-NMR (CDCl_3,_ 150 MHz, δ/ppm): 162.68 (4’), 157.98 (5’“‘), 148.07 (3’“‘), 138.88 (1’‘), 130.77 (5’‘), 130.70 (4’‘), 130.47 (2’‘), 129.89 (3’‘), 129.70 (2’, 6’), 129.26 (6’‘), 126.79 (1’), 114.49 (3’, 5’), 55.69 (1’“), 45.42 (2,6), 35.65 (3’“), 30.46 (4), 29.65 (2’“), 28.81 (3,5).

***3-[(3-Bromobenzyl)thio]-5-{1-[(4-methoxypheneyl)sulfonyl]-4-piperidinyl}-4-methyl-4H-1,2,4-triazole (7i).*** Off white amorphous solid; yield: 90%; M.P.: 153.0^o^C; HRMS: [M]^•+^ 536.0544 (Calcd. for C_22_H_25_BrN_4_O_3_S_2_; 536.0551); Anal. Calcd. for C_22_H_25_BrN_4_O_3_S_2_: C, 49.16; H, 4.69; Br, 14.87; N, 10.42; O, 8.93; S, 11.93; found C, 49.12; H, 4.65; Br, 14.83; N, 10.40; O, 8.89; S, 11.90; IR (KBr, υ_*max*_, cm^-1^): 2800 (Aromatic carbon hydrogen single bond), 1610 (Carbon nitrogen double bond), 1500 (Aromatic carbon double bond), 1330 (Methyl group), 1300 (Sulphur monoxide), 1200 (Carbon oxygen single bond), 659 (Carbon bromide); ^1^H-NMR (CDCl_3,_ 600 MHz, δ/ppm) (coupling constant, *J* = Hertz): 7.70 (d, coupling constant = 8.8 Hz, 2H, 2’, 6’), 7.44 (d, coupling constant = 7.9 Hz, 1H, 4’‘), 7.31-7.22 (m, 3H, 2’‘, 5’‘, 6’‘), 7.17 (d, coupling constant = 8.8 Hz, 2H, 3’, 5’), 4.29 (s, 2H, 3’“), 3.86 (s, 3H, 1’“), 3.62–3.60 (m, 2H, 2e, 6e), 3.20 (s, 3H, 2’“), 2.80–2.77 (m, 1H, 4), 2.41–2.38 (m, 2H, 2a, 6a), 1.89–1.86 (m, 2H, 3e, 5e), 1.73–1.67 (m, 2H, 3a, 5a); ^13^C-NMR (CDCl_3,_ 150 MHz, δ/ppm): 162.68 (4’), 158.02 (5’“‘), 147.86 (3’“‘), 134.64 (3’‘), 133.04 (1’‘), 131.24 (2’‘), 129.70 (2’, 6’), 129.53 (4’‘), 129.41 (5’‘), 127.25 (6’‘), 126.80 (1’), 114.49 (3’, 5’), 55.70 (1’“), 45.42 (2,6), 35.62 (3’“), 30.51 (4), 29.51 (2’“), 28.80 (3, 5).

***3-((4-Fluorobenzyl)thio)-5-{1-((4-methoxyphenyl)sulfonyl)-4-piperidinyl}-4-methyl-4H-1,2,4-triazole (7j).*** White amorphous solid; yield: 91%; M.P: 191.2^o^C; HRMS: (M)^•+^ 476.1343 (Calcd. for C_22_H_25_FN_4_O_3_S_2_; 476.1352); Anal. Calcd. for C_22_H_25_FN_4_O_3_S_2_: C, 55.44; H, 5.29; F, 3.99; N, 11.76; O, 10.07; S, 13.46; found C, 55.40; H, 5.25; F, 3.94; N, 11.70; O, 10.02; S, 13.40; IR (KBr, υ_*max*_, cm^-1^): 2800 (Aromatic carbon hydrogen single bond), 1600 (Carbon nitrogen double bond), 1510 (Aromatic carbon double bond), 1320 (Methyl group), 1310 (Sulphur monoxide), 1200 (Carbon oxygen single bond), 1037 (Carbon fluoride); ^1^H-NMR (CDCl_3,_ 600 MHz, δ/ppm) (coupling constant, *J* = Hertz): 7.70 (d, coupling constant = 8.8 Hz, 2H, 2', 6'), 7.28-7.25 (m, 2H, 3'', 5''), 7.18 (d, coupling constant = 8.8 Hz, 2H, 3', 5'), 7.11-7.08 (m, 2H, 2'', 6''), 4.24 (s, 2H, 3'''), 3.86 (s, 3H, 1'''), 3.62-3.60 (m, 2H, 2e, 6e), 3.23 (s, 3H, 2'''), 2.81-2.77 (m, 1H, 4), 2.41-2.37 (m, 2H, 2a, 6a), 1.90-1.87 (m, 2H, 3e, 5e), 1.73-1.67 (m, 2H, 3a, 5a); ^13^C-NMR (CDCl_3,_ 150 MHz, δ/ppm): 162.68 (4'), 160.60 (4''), 157.85 (5''''), 148.37 (3''''), 133.61 (1''), 130.94 (3'', 5''), 129.70 (2', 6'), 126.78 (1'), 115.22 (2'', 6''), 114.49 (3', 5'), 55.69 (1'''), 45.44 (2, 6), 36.50 (3'''), 30.49 (4), 29.56 (2'''), 28.83 (3, 5).

### 3.3 Biological activity determination

#### 3.3.1 AChE and BChE assays.

The biological evaluation of whole series of synthesized against AChE and BChE was carried out by following the pre-reported methodology with little modifications [47]. The chemical makeup of the chemical reaction is shown in the equation 3 below, with eserine serving as the standard.


$$Acetylcholine\;+\;H_{\text{2}}O\overset{AChE,BChE}{\to }Thiocholine\;+\;acetic\;acid$$ (3)

$$Thiocholine\;+\;DTNB\underset{\left[Yellow\;Color,\;410\;nm\right]}{\to }Formation\;of\;TNB$$

The reaction mixture in 100 μL comprised test solutions, the corresponding enzyme AChE or BChE, and 60 μL of pH 7.7 phosphate buffer. Following a 5-minute pre-incubation period at 37 °C, 405 nm absorbance was calculated. Ten microliters of 0.5 mM DTNB and 0.5 mM of either 10 μL butyrylthiocholine chloride (for BChE) or 0.5 mM of acetylthiocholine (for AChE) were added to initiate the reaction. Using a 96-well plate sensor (Synergy HT, BioTek, USA), the contents were measured at 405 nm after 20–25 minutes of incubation at 37 ºC. The provided formula [**4**] was used to compute the inhibition (%).


$$\text{Inhibition }(\%)=\frac{\text{Control}-\text{Test}}{\text{Control}}\times \text{100}$$ (4)

Active compounds’s IC_50_ values were evaluated utilizing program named Ez-Fit (Perrella Scientific Inc., Amherst, USA).

#### 3.3.2 15-LOX assay.

UV-based method was used to examine the synthetic compounds’ ability to block 15-LOX enzyme, as earlier described [48]. In the 96-well plate, a reaction mixture comprising the test chemical, pH 6.8 buffer solution, and 15-LOX were prepared. At 234 nm, the absorbance was measured following a 5-minute pre-incubation. The combination was first allowed to react for 10 minutes by incubating it with twenty-five micro liter solution of substrate of linoleic acid. At 234 nm, the absorbance was measured following incubation. Each experiment was conducted thrice. IC_50_ values of active substances were evaluated using the cholinesterase enzymes mentioned above.

#### 3.3.3 An assay on α-Glucosidase inhibition.

α-glucosidase test was performed employing Pierre’s method [49]. Ten minutes were spent incubating the combination at 37 °C after test sample (10 µL) and α-glucosidase inhibitor (10 µL) were added to a pH 6.8 phosphate buffer. Two measurements of the absorbance were made: one around 400 nm and the other after an incubation time of twenty minutes. Acarbose was used as the standard medication for all three test runs. The inhibition % was determined using Equation 4. Equation 4 states that the absorbance during the presence of the test sample is called the test, and the absorbance during its absence is called the control.

### 3.4 Molecular docking studies

The docking studies yielded information on the interactions between chemicals and an enzyme’s active site, i.e., inhibitory mechanism and the criteria by which compounds and enzymes interact [50]. Using SYBYL-X 1.3, the structures of some proteins that were known were developed. Using the AMBER 7 FF99 force field, the missing hydrogen and diversity of atoms were obtained. Using a Tripo force field and an atomic charge of GasteigereHückel, SYBYL-X 1.3 was applied to generate the inhibitors’ 3-D architectures. Modifications to the Surflex-docked module of SYBYL-X1.3 were made using the technique that was disclosed [51]. Molecular docking is a computational technique employed to predict the most advantageous binding conformations of piperidine-based thiazole analogues against acetyl cholinesterase (ACHE), butyrylcholinesterase1 (BCHE), 15-lipoxyenase (15-LOX) and α-Glucosidase. All piperidine-based thiazole analogues (**7a-j**) compounds were selected for docking studies. Donepezil co-crystal structure of ACHE (4EY7), N-((1-benzylpiperidin-3-yl)methyl)-N-(2-methoxyethyl)naphthalene-2-sulfonamide co-crystal structure of BCHE (5DYW), co-crystal structure of 15-LOX (4NRE) were acquired from the Protein Data Bank (PDB). Since no co-crystallized 3D structures for *Saccharomyces cerevisiae* α-glucosidase are available in the Protein Data Bank (PDB), the FASTA sequence (NCBI Accession No. **P07265**) was retrieved from UniProt and modeled using the Phyre2 server. The generated structure with the highest confidence was rigorously validated through PROCHECK Ramachandran plot analysis and ProSAweb Z-score evaluation to ensure stereochemical and structural reliability. This modeled α-glucosidase has also been validated and employed in our previous investigations, where it served as a reliable framework for docking and virtual screening, further supporting its suitability for the present study [52].

The Protein Preparation Wizard program was used to create all proteins in Schrödinger version 2020−3. The structure was completed by incorporating the absent side chains and loops using Prime. Hydrogen was added to the compound and amino acid residues after any original hydrogen atoms were removed. The protonation and tautomeric states of Asp, Glu, Arg, Lys, and His were adjusted to a pH of 7.4 using ProPka. Following the removal of water molecules from the active site that were more than 5.0 Å from the ligand, the protein configuration was minimized and optimized. Then, using OPLS4 force field restrained minimization with heavy atom convergence; the protein-ligand complex was geometrically refined. The sitemap module was used to perform the binding site analysis. All of the ligand molecules were created using the LigPrep wizard program.

With a given input structure, this program can create a variety of compounds with different ionization states, stereochemical characteristics of tautomers, and ring conformations. The ligands were optimized to have the lowest energy conformations using the OPLS4 force field. It was concluded that building a grid within a 10-crystallized compound’s radius required the use of a glide grid module. Glide receptor grids are created using the grid development process based on ligand-binding sites. The term “Glide receptor grid” describes a defined area of investigation that includes data regarding the force field surrounding the receptor protein. At the active site, the glide XP (extra precision) mode was utilized to complete the docking procedure. The docking computation yielded a top-scored predicted complex, which was then assessed by the scoring function.

To visualize and comprehensively annotate the docking poses, including hydrogen bonds, hydrophobic interactions, π–π stacking, and other non-covalent contacts, the Protein–Ligand Interaction Profiler (PLIP) online tool was employed. This ensured that all relevant interactions within the binding site were clearly captured for figure generation and analysis.

### 3.5 Structural interaction fingerprinting analysis

The novel method, SIFt (Structural Interaction Fingerprint), translates 3D protein-ligand binding interactions into a one-dimensional binary string representing the structural interaction profile. This fingerprint aids in organizing, analyzing, and visualizing complex ligand-receptor interactions. SIFt has been applied in three areas of structure-based drug design: clustering docking poses with similar binding modes, analyzing ∼90 X-ray protein kinase-inhibitor complexes, and filtering virtual chemical libraries to select molecules with desirable binding interactions. SIFt uses structural data for drug design in an efficient manner. Analyzing each compound’s (**7a–7j**) interaction patterns separately can be difficult and complex. To overcome this, we created a SIFt map that will enable a more accurate interpretation of the interaction patterns of the hits that are being studied. Researchers can gain a better understanding of how large ligand databases bind to a protein’s active site by using a well-constructed Structure Interaction Fingerprint (SIFt). In order to determine the main hotspot residues involved in compound-protein interactions, we created a SIFt in this study to analyze the binding patterns of all compounds (**7a-7j**) hits binding to ACHE, BCHE, 15-LOX, and α-glucosidase aiming to identify the primary hotspot residues involved in compound-protein complex formation.

### 3.6 Toxicological modelling and ADME profiling

The total clearance rates, a measure of the drugs elimination from the body, are crucial for determining appropriate dosing to achieve steady-state concentrations. This ADME study provides total clearance rates in terms of log (ml/min/kg), aiding in the optimization of therapeutic dosages. The metabolism profiles of these compounds suggest interactions with CYP3A4, indicating their metabolic pathway involves this significant enzyme, which could influence drug-drug interactions. The total clearance rates, a measure of the drugs elimination from the body, are crucial for determining appropriate dosing to achieve steady-state concentrations. Present analysis, employing tools like the pkCSM web application, has facilitated a detailed evaluation of these compounds, displaying their therapeutic potential. To evaluate the in silico ADMET properties of these molecules, the pkCSM online tool (https://biosig.lab.uq.edu.au/pkcsm/prediction) was employed. When a drug is administered orally, the primary site of absorption is typically the digestive tract. The extent to which a substance is taken up by the human gastrointestinal tract is influenced by various parameters related to intestinal absorption. When the absorption rate of a molecule falls below 30%, it is considered to exhibit inadequate absorption efficacy (source: https://biosig.lab.uq.edu.au/pkcsm/theory). On the other hand, other compounds show relatively lower levels of intestinal absorption, suggesting they might have limitations in their ability to pass through the intestinal barrier and get absorbed into the body’s circulation.

Cytochrome P450 (CYP450) is a hepatic enzyme primarily responsible for the oxidation of xenobiotic, aiding in their elimination. Numerous medications can be either activated or deactivated by various isoforms of cytochrome P450, such as CYP1A2, CYP2C19, CYP2C9, and CYP2D6. Therefore, maximizing the inhibitory potential of a chemical compound is crucial for effectively blocking the enzyme. Cytochrome P450 enzymes play a crucial role in the processing of many drugs. However, inhibitors of P450 enzymes can significantly alter the way these compounds are metabolized, affecting their pharmacokinetics. Therefore, it is important to consider that the chemicals provided are likely substrates of cytochrome P450. Among the various isoforms, cytochrome P450 3A4 is primarily responsible for drug metabolism. The results in the table indicate whether the substances listed will undergo metabolism by cytochrome P450 or not. Bioavailability is a crucial factor in determining appropriate dosage rates to achieve steady-state concentration. Table also presents the logarithmic values of expected total clearance (CLtot) of a drug measured in (ml/min/kg). The AMES toxicity test is commonly employed to assess a compound’s potential for inducing bacterial mutations. A positive result suggests that the material is mutagenic, which means it will function similarly to a carcinogenic agent.

### 3.7 MESP studies and DFT

MESP analyses were conducted in aqueous environments to assess the biological effects of all compounds under physiological conditions, offering a relevant scenario for the evaluation of these potential therapeutics. The MESP mappings accentuate regions of high electronegative potential, marked by a deep red color across all four structures. These critical areas suggest favorable sites for electrophilic attack, which is vital for effective molecular binding and inhibition of all enzymes. Employing Koopmans’s theorem, global reactivity parameters for compound **7h** and **7i**, have been computed, demonstrating notable differences in their electronic properties as depicted in the data.

### 3.8 Dynamic simulations, comprehensive analysis of structural flexibility and stability

Molecular Dynamics (MD) simulations function as a computational tool that provides insights into molecular interactions at the atomic level. They can be used to discern protein configurations and movements, drug binding to particular targets, and the intricate interactions between molecular components in ligand-protein bonded systems. To investigate the binding mechanisms and interaction modes of inhibitors with different affinities towards α-glucosidase and 15-LOX, molecular dynamics (MD) simulations were performed in conjunction with free energy calculations. Root-mean-square deviation (RMSD) to the benchmark compound **7h** across 100 ns of molecular dynamics (MD) simulation was used to assess the dynamic stability of systems and validate the sampling technique. Root Mean Square Fluctuation (RMSF) values of central amino acid residues in the LOX-**7h** complex system and α-glucosidase-**7h** complex system were analyzed to determine their flexibility patterns. RMSF values, which indicate the levels of flexibility, show that higher values correlate with increased flexibility, while lower values suggest less flexibility.

### 3.9 Binding free energy analysis

The MM/PBSA and MM/GBSA techniques were utilized to ascertain the selected compound 7h binding affinities to LOX and α-glucosidase. While the binding free energies calculated with MM/PBSA and MM/GBSA were marginally higher than the absolute values obtained from experiments (ΔGexp).

### 3.10 Protein binding studies

The bioactive effectiveness of synthesized compounds could be justified based on the interactions of drug candidates with blood protein serum albumin. Delivery of the drugs to the site interaction could be understood by knowing the mode of binding of compounds with drugs. The strong or weak binding interactions affect drug delivery. Fluorescent investigations are the most preferred and accurate way to determine the compound-albumin interaction. Using fluorometric calibration, a buffer solution containing phosphate (3 mL, 3 M) and synthetic chemical solutions (1 mg per milliliter in DMSO solvent) were tested to determine BSA quenching constant. Following the stimulation of the solutions at 295 nm, the BSA solutions’ intensity both with and without test compounds was determined at 298 K at 336 nm. For the purpose of performing thermodynamic evaluations of BSA and its produced derivatives, respectively, experiments were conducted on all the generated compounds at two additional temperatures, 303 K and 308 K. As consequently, fluorescence spectra’s 3 sets at 303, 308, and 298 K were acquired. In order to perform site-dependent binding experiments, BSA solution (three mL, 3.0 M) containing and excluding site identifiers (warfarin or ibuprofen) was titrated fluorometrically employing mixtures of generated compounds (one mg per milliliter in DMSO). The solutions were excited by scanning them at 295 nm wavelength at 298 K. Either the site markers or each of the produced series of triazole derivatives were titrated in BSA solution (3 mL, 3 M) at a wavelength of stimulation of 295 nm for the synchronous binding assays (at 15 nm or 60 nm) [53].

### Analytical statistics

Microsoft Excel 2010 was used to conduct statistical assessments, with each calculation being done three times. The findings were displayed as Mean ± SEM. The EZ-Fit Enzyme Kinetics Program (Perrella Scientific Inc., Amherst, USA) was used to calculate the IC_50_ values.

## 4 Conclusion

A series of 10 compounds bearing heterocyclic moieties was synthesized through traditional and microwave assisted methods. Microwave assisted technique proved outstanding regarding reaction time and percent yield. Spectral studies through IR, HNMR and CNMR justified the molecular structures. The most active compounds **7h, 7c** and **7i** presented good inhibition potential against AChE enzyme while compound **7i** was found most active against BChE. Compounds **7i** and **7e** displayed excellent potential against 15-LOX inhibitory profiles while Whole series found highly active against α-Glucosidase with variable range. Molecular docking studies supported the *in vitro* profiles of enzyme inhibition. Studies on BSA interaction clearly presented the binding interactions of five compounds with this protein. Further work is continued against most potent enzyme inhibitors for further evaluation.

## Supporting information

S1 Fig ^13^CNMR spectrum of 3-benzylthio-5-{1-[(4-methoxyphenyl)sulfonyl]-4-piperidinyl}-4-methyl-4*H*-1,2,4-triazole (7a). (DOCX)

S2 Fig ^1^HNMR spectrum of 3-benzylthio-5-{1-[(4-methoxyphenyl)sulfonyl]-4-piperidinyl}-4-methyl-4*H*-1,2,4-triazole (7a). (DOCX)

S3 Fig ^13^CNMR spectrum of 3-[(2-methylbenzyl)thio]-5-{1-[(4-mthoxyphenyl)soulfonyl]-4-piperidinyl}-4-methyl-4*H*-1,2,4-triazole (7b). (DOCX)

S4 Fig ^1^HNMR spectrum of 3-[(2-methylbenzyl)thio]-5-{1-[(4-mthoxyphenyl)soulfonyl]-4-piperidinyl}-4-methyl-4*H*-1,2,4-triazole (7b). (DOCX)

S5 Fig ^13^CNMR spectrum of 3-[(3-methylbenzyl)thio]-5-{1-[(4-methoxyphenyl)sulfonyl]-4-piperidinyl}-4-methyl-4*H*-1,2,4-triazole (7c). (DOCX)

S6 Fig ^1^HNMR spectrum of 3-[(3-methylbenzyl)thio]-5-{1-[(4-methoxyphenyl)sulfonyl]-4-piperidinyl}-4-methyl-4*H*-1,2,4-triazole (7c). (DOCX)

S7 Fig ^13^CNMR spectrum of 3-[(4-methylbenzyl)thio]-5-{1-[(4-methoxyphenyl)sulfonyl]-4-piperidinyl}-4-methyl-4*H*-1,2,4-triazole (7d). (DOCX)

S8 Fig ^1^HNMR spectrum of 3-[(4-methylbenzyl)thio]-5-{1-[(4-methoxyphenyl)sulfonyl]-4-piperidinyl}-4-methyl-4*H*-1,2,4-triazole (7d). (DOCX)

S9 Fig ^13^CNMR spectrum of 3-[(2-chlorobenzyl)thio]-5-{1-[(4-methoxyphenyl)sulfonyl]-4-piperidinyl}-4-methyl-4*H*-1,2,4-triazole (7e). (DOCX)

S10 Fig ^1^HNMR spectrum of 3-[(2-chlorobenzyl)thio]-5-{1-[(4-methoxyphenyl)sulfonyl]-4-piperidinyl}-4-methyl-4*H*-1,2,4-triazole (7e). (DOCX)

S11 Fig ^13^CNMR spectrum of 3-[(4-chlorobenzyl)thio]-5-{1-[(4-methoxyphenyl)sulfonyl]-4-piperidinyl}-4-methyl-4*H*-1,2,4-triazole (7f). (DOCX)

S12 Fig ^1^HNMR spectrum of 3-[(4-chlorobenzyl)thio]-5-{1-[(4-methoxyphenyl)sulfonyl]-4-piperidinyl}-4-methyl-4*H*-1,2,4-triazole (7f). (DOCX)

S13 Fig ^13^CNMR spectrum of 3-[(2,4-dichlorobenzyl)thio]-5-{1-[(4-methoxyphenyl) sulfonyl]-4-piperidinyl}-4-methyl-4*H*-1,2,4-triazole (7g). (DOCX)

S14 Fig ^1^HNMR spectrum of 3-[(2,4-dichlorobenzyl)thio]-5-{1-[(4-methoxyphenyl) sulfonyl]-4-piperidinyl}-4-methyl-4*H*-1,2,4-triazole (7g). (DOCX)

S15 Fig ^13^CNMR spectrum of 3-[(3,4-dichlorobenzyl)thio]-5-{1-[(4-methoxyphenyl) sulfonyl]-4-piperidinyl}-4-methyl-4*H*-1,2,4-triazole (7h). (DOCX)

S16 Fig ^1^HNMR spectrum of 3-[(3,4-dichlorobenzyl)thio]-5-{1-[(4-methoxyphenyl) sulfonyl]-4-piperidinyl}-4-methyl-4*H*-1,2,4-triazole (7h). (DOCX)

S17 Fig ^13^CNMR spectrum of 3-[(3-bromobenzyl)thio]-5-{1-[(4-methoxypheneyl)sulfonyl]-4-piperidinyl}-4-methyl-4*H*-1,2,4-triazole (7i). (DOCX)

S18 Fig ^1^HNMR spectrum of 3-[(3-bromobenzyl)thio]-5-{1-[(4-methoxypheneyl)sulfonyl]-4-piperidinyl}-4-methyl-4*H*-1,2,4-triazole (7i). (DOCX)

S19 Fig ^13^CNMR spectrum of 3-[(4-fluorobenzyl)thio]-5-{1-[(4-methoxyphenyl)sulfonyl]-4-piperidinyl}-4-methyl-4*H*-1,2,4-triazole (7j). (DOCX)

S20 Fig ^1^HNMR spectrum of 3-[(4-fluorobenzyl)thio]-5-{1-[(4-methoxyphenyl) sulfonyl]-4-piperidinyl}-4-methyl-4H-1,2,4-triazole (7j). (DOCX)

## References

[1] Mingos DMP, Baghurst DR. Tilden Lecture. Applications of microwave dielectric heating effects to synthetic problems in chemistry. Chem Soc Rev. 1991;20:1.

[2] Lidström P, Tierney J, Wathey B, Westman J. Microwave assisted organic synthesis—a review. Tetrahedron. 2001;57(45):9225–83. doi: 10.1016/s0040-4020(01)00906-1

[3] Loupy A, Wiley-VCH Weinheim. Microwaves in Organic Synthesis Edited by Andre Loupy, Wiley-VCH: Weinheim. 2002.

[4] Kappe CO. Controlled microwave heating in modern organic synthesis. Angew Chem Int Ed Engl. 2004;43(46):6250–84. doi: 10.1002/anie.200400655 15558676

[5] Kappe CO, Stadler A. Weinheim: Wiley-VCH. 2005.

[6] Virk NA, Rehman A, Abbasi MA, Siddiqui SZ, Iqbal J, Rasool S, et al. Microwave‐assisted synthesis of triazole derivatives conjugated with piperidine as new anti‐enzymatic agents. Journal of Heterocyclic Chem. 2019;57(3):1387–402. doi: 10.1002/jhet.3875

[7] Strauss CR, Trainor RW. Developments in microwave-assisted organic chemistry. Aust J Chem. 1995;48:1665–92.

[8] Larhed M, Moberg C, Hallberg A. Microwave-accelerated homogeneous catalysis in organic chemistry. Acc Chem Res. 2002;35(9):717–27. doi: 10.1021/ar010074v 12234201

[9] Leadbeater NE. Fast, easy, clean chemistry by using water as a solvent and microwave heating: the Suzuki coupling as an illustration. Chem Commun. 2005;23:2881–902. 10.1039/b500952a 15957019

[10] Roberts BA, Strauss CR. Toward rapid, “green”, predictable microwave-assisted synthesis. Acc Chem Res. 2005;38(8):653–61. doi: 10.1021/ar040278m 16104688

[11] Javid J, Aziz‐ur‐Rehman, Abbasi MA, Siddiqui SZ, Iqbal J, Virk NA, et al. Comparative conventional and microwave assisted synthesis of heterocyclic oxadiazole analogues having enzymatic inhibition potential. Journal of Heterocyclic Chem. 2020;58(1):93–110. 10.1002/jhet.4150

[12] Stefani HA, Vasconcelos SNS, Souza FB, Manarin F, Zukerman-Schpector J. One-pot three-component synthesis of indole-3-glyoxyl derivatives and indole-3-glyoxyl triazoles. Tetrahedron Letters. 2013;54(43):5821–5. doi: 10.1016/j.tetlet.2013.08.064

[13] Yue H, Zhu C, Kancherla R, Liu F, Rueping M. Regioselective Hydroalkylation and Arylalkylation of Alkynes by Photoredox/Nickel Dual Catalysis: Application and Mechanism. Angew Chem Int Ed Engl. 2020;59(14):5738–46. doi: 10.1002/anie.201914061 31901214 PMC7154703

[14] Dömling A, Wang W, Wang K. Chemistry and biology of multicomponent reactions. Chem Rev. 2012;112(6):3083–135. doi: 10.1021/cr100233r 22435608 PMC3712876

[15] Ruijter E, Orru R, van der Heijden G. Efficiency, Diversity, and Complexity with Multicomponent Reactions. Synlett. 2013;24(06):666–85. doi: 10.1055/s-0032-1318222

[16] Dedola S, Nepogodiev SA, Field RA. Recent applications of the Cu(I)-catalysed Huisgen azide-alkyne 1,3-dipolar cycloaddition reaction in carbohydrate chemistry. Org Biomol Chem. 2007;5(7):1006–17. doi: 10.1039/b618048p 17377651

[17] Dondoni A. Triazole: the keystone in glycosylated molecular architectures constructed by a click reaction. Chem Asian J. 2007;2(6):700–8. doi: 10.1002/asia.200700015 17464957

[18] Aragão-Leoneti V, Campo VL, Gomes AS, Field RA, Carvalho I. Application of copper(I)-catalysed azide/alkyne cycloaddition (CuAAC) ‘click chemistry’ in carbohydrate drug and neoglycopolymer synthesis. Tetrahedron. 2010;66(49):9475–92. doi: 10.1016/j.tet.2010.10.001

[19] Dedola S, Hughes DL, Nepogodiev SA, Rejzek M, Field RA. Synthesis of alpha- and beta-D-glucopyranosyl triazoles by CuAAC “click chemistry”: reactant tolerance, reaction rate, product structure and glucosidase inhibitory properties. Carbohydr Res. 2010;345(9):1123–34. doi: 10.1016/j.carres.2010.03.041 20427038

[20] Ye N, Chen H, Wold EA, Shi P-Y, Zhou J. Therapeutic Potential of Spirooxindoles as Antiviral Agents. ACS Infect Dis. 2016;2(6):382–92. doi: 10.1021/acsinfecdis.6b00041 27627626 PMC5417367

[21] Haddad S, Boudriga S, Akhaja TN, Raval JP, Porzio F, Soldera A, et al. A strategic approach to the synthesis of functionalized spirooxindole pyrrolidine derivatives: in vitro antibacterial, antifungal, antimalarial and antitubercular studies. New J Chem. 2015;39(1):520–8. doi: 10.1039/c4nj01008f

[22] Musad EA, Mohamed R, Saeed BA, Vishwanath BS, Rai KL. Synthesis and evaluation of antioxidant and antibacterial activities of new substituted bis (1,3,4-oxadiazoles), 3,5-bis (substituted) pyrazoles and isoxazoles. Bioorg Med Chem Lett. 2011;21:3536–40. 21612921 10.1016/j.bmcl.2011.04.142

[23] Jha KK, Samad A, Kumar Y, Shaharyar M, Khosa RL, Jain J, et al. Design, synthesis and biological evaluation of 1,3,4-oxadiazole derivatives. Eur J Med Chem. 2010;45(11):4963–7. doi: 10.1016/j.ejmech.2010.08.003 20817328

[24] Ramaprasad GC, Kalluraya B, Kumar BS, Hunnur RK. Synthesis and biological property of some novel 1,3,4-oxadiazoles. Eur J Med Chem. 2010;45(10):4587–93. doi: 10.1016/j.ejmech.2010.07.021 20708305

[25] Rashid M, Husain A, Mishra R. Synthesis of benzimidazoles bearing oxadiazole nucleus as anticancer agents. Eur J Med Chem. 2012;54:855–66. doi: 10.1016/j.ejmech.2012.04.027 22608854

[26] Cummings J, Lee G, Nahed P, Kambar MEZN, Zhong K, Fonseca J, et al. Alzheimer’s disease drug development pipeline: 2022. Alzheimers Dement (N Y). 2022;8(1):e12295. doi: 10.1002/trc2.12295 35516416 PMC9066743

[27] Dos Santos Picanco LC, Ozela PF, de Fatima de Brito Brito M, Pinheiro AA, Padilha EC, Braga FS, et al. Alzheimer’s Disease: A Review from the Pathophysiology to Diagnosis, New Perspectives for Pharmacological Treatment. Curr Med Chem. 2018;25(26):3141–59. doi: 10.2174/0929867323666161213101126 30191777

[28] Pohanka M. Cholinesterases, a target of pharmacology and toxicology. Biomed Pap Med Fac Univ Palacky Olomouc Czech Repub. 2011;155(3):219–29. doi: 10.5507/bp.2011.036 22286807

[29] Estrada-Valencia M, Herrera-Arozamena C, Pérez C, Viña D, Morales-García JA, Pérez-Castillo A, et al. New flavonoid - N,N-dibenzyl(N-methyl)amine hybrids: Multi-target-directed agents for Alzheimer´s disease endowed with neurogenic properties. J Enzyme Inhib Med Chem. 2019;34(1):712–27. doi: 10.1080/14756366.2019.1581184 31852270 PMC6407579

[30] Vishnupriya P, Aparna A, Viswanadha VP. Lipoxygenase (LOX) Pathway: A Promising Target to Combat Cancer. Curr Pharm Des. 2021;27(31):3349–69. doi: 10.2174/1381612826666210101153216 33388012

[31] Chen L, Deng H, Cui H, Fang J, Zuo Z, Deng J, et al. Inflammatory responses and inflammation-associated diseases in organs. Oncotarget. 2017;9(6):7204–18. doi: 10.18632/oncotarget.23208 29467962 PMC5805548

[32] Hu C, Ma S. Recent development of lipoxygenase inhibitors as anti-inflammatory agents. Medchemcomm. 2017;9(2):212–25. doi: 10.1039/c7md00390k 30108915 PMC6083793

[33] Green GA. Understanding NSAIDs: from aspirin to COX-2. Clin Cornerstone. 2001;3(5):50–60. doi: 10.1016/s1098-3597(01)90069-9 11464731

[34] Lu P, Schrag ML, Slaughter DE, Raab CE, Shou M, Rodrigues AD. Mechanism-based inhibition of human liver microsomal cytochrome P450 1A2 by zileuton, a 5-lipoxygenase inhibitor. Drug Metab Dispos. 2003;31(11):1352–60. doi: 10.1124/dmd.31.11.1352 14570767

[35] Wang X, Wu Y, Wang ZY, Li JJ, Li W, Li Q, et al. Anti-inflammatory iridoid glycosides from fruits of Cornus officinalis. Phytochem Lett. 2022;52:122–5.

[36] Tian RD, Sheng MK, Zhao YF, Wen CN, Liu M, Ma BJ. α-Pyrone derivatives from the endophytic Fusarium sp. L33 isolated from Dioscorea opposite. Phytochem Lett. 2024;62:14–7.

[37] Wang Q, Gao F, Chen X, Wu W, Wang L, Shi J, et al. Characterization of key aroma compounds and regulation mechanism of aroma formation in local Binzi (Malus pumila × Malus asiatica) fruit. BMC Plant Biol. 2022;22(1):532. doi: 10.1186/s12870-022-03896-z 36380276 PMC9664629

[38] Cheung J, Rudolph MJ, Burshteyn F, Cassidy MS, Gary EN, Love J, et al. Structures of human acetylcholinesterase in complex with pharmacologically important ligands. J Med Chem. 2012;55(22):10282–6. doi: 10.1021/jm300871x 23035744

[39] Košak U, Brus B, Knez D, Šink R, Žakelj S, Trontelj J, et al. Development of an in-vivo active reversible butyrylcholinesterase inhibitor. Sci Rep. 2016;6:39495. doi: 10.1038/srep39495 28000737 PMC5175178

[40] Kobe MJ, Neau DB, Mitchell CE, Bartlett SG, Newcomer ME. The structure of human 15-lipoxygenase-2 with a substrate mimic. J Biol Chem. 2014;289(12):8562–9. doi: 10.1074/jbc.M113.543777 24497644 PMC3961679

[41] Virk NA, Iqbal J, Rehman AU, Abbasi MA, Siddiqui SZ, Rasool S, et al. In vitro biological assessment of 1,3,4-oxadiazole sandwiched by azinane and acetamides supported by molecular docking and BSA binding studies. Curr Chem Lett. 2023;12:353–68.

[42] Dedola S, Nepogodiev SA, Field RA. Recent applications of the Cu(I)-catalysed Huisgen azide-alkyne 1,3-dipolar cycloaddition reaction in carbohydrate chemistry. Org Biomol Chem. 2007;5(7):1006–17. doi: 10.1039/b618048p 17377651

[43] Deng T, Zhao J, Peng D, He X, Huang X-A, Lin C, et al. Probing the serum albumin binding site of fenamates and photochemical protein labeling with a fluorescent dye. Org Biomol Chem. 2022;20(25):5076–85. doi: 10.1039/d2ob00717g 35697330

[44] Virk NA, Rehman AU, Shuaib A, Iqbal J, Rasool S, Al-Mijalli SH, et al. Novel 1,2,4-triazoles as anti-enzymatic agents: Microwave versus conventional synthesis, characterization, docking and BSA binding studies. J Mol Struct. 2023;1281:135070.

[45] Zhang Y, Zhou W, Xu N, Wang G, Li J, An K, et al. Aniline as a TICT rotor to derive methine fluorogens for biomolecules: A curcuminoid-BF2 compound for lighting up HSA/BSA. Chin Chem Lett. 2023;34:107472.

[46] Virk NA, Rehman A, Abbasi MA, Siddiqui SZ, Rashid U, Iqbal J, et al. Conventional versus microwave assisted synthesis, molecular docking and enzyme inhibitory activities of new 3,4,5-trisubstituted-1,2,4-triazole analogues. Pak J Pharm Sci. 2018;31(4(Supplementary)):1501–10. 30058542

[47] Ellman GL, Courtney KD, Andres V Jr, Feather-Stone RM. A new and rapid colorimetric determination of acetylcholinesterase activity. Biochem Pharmacol. 1961;7:88–95. doi: 10.1016/0006-2952(61)90145-9 13726518

[48] Shahid W, Ejaz SA, Al-Rashida M, Saleem M, Ahmed M, Rahman J, et al. Identification of NSAIDs as lipoxygenase inhibitors through highly sensitive chemiluminescence method, expression analysis in mononuclear cells and computational studies. Bioorg Chem. 2021;110:104818. doi: 10.1016/j.bioorg.2021.104818 33784531

[49] Tappel AL. The mechanism of the oxidation of unsaturated fatty acids catalyzed by hematin compounds. Arch Biochem Biophys. 1953;44(2):378–95. doi: 10.1016/0003-9861(53)90056-3 13058395

[50] Ghersi D, Sanchez R. Beyond structural genomics: computational approaches for the identification of ligand binding sites in protein structures. J Struct Funct Genomics. 2011;12(2):109–17. doi: 10.1007/s10969-011-9110-6 21537951 PMC3127736

[51] Jain AN. Surflex-Dock 2.1: robust performance from ligand energetic modeling, ring flexibility, and knowledge-based search. J Comput Aided Mol Des. 2007;21(5):281–306. doi: 10.1007/s10822-007-9114-2 17387436

[52] Khalid MF, Rehman K, Irshad K, Chohan TA, Akash MSH. Biochemical Investigation of Inhibitory Activities of Plant-Derived Bioactive Compounds Against Carbohydrate and Glucagon-Like Peptide-1 Metabolizing Enzymes. Dose Response. 2022;20(2):15593258221093275. doi: 10.1177/15593258221093275 35574252 PMC9099060

[53] Wu X, Liu J, Huang H, Xue W, Yao X, Jin J. Interaction studies of aristolochic acid I with human serum albumin and the binding site of aristolochic acid I in subdomain IIA. Int J Biol Macromol. 2011;49(3):343–50. doi: 10.1016/j.ijbiomac.2011.05.010 21635915

