# Supplementary material for: Structural engineering, BSA binding and computational analysis of isonipacotate based enzyme inhibitors containing 1,2,4-triazole
Source: PLoS One. 2026 Jan 7;21(1):e0337642. doi: 10.1371/journal.pone.0337642 (PMC12779149; doi:10.1371/journal.pone.0337642)
Supplement: S18 Fig — (DOCX) [file pone.0337642.s018.docx]

**Structural Engineering, BSA Binding and Computational Analysis of Isonipacotate Based Enzyme Inhibitors Containing 1,2,4-Triazole**

| \|  \| \| --- \| |
| --- | --- |

| \| \|  \| \| --- \| \| \| --- \| --- \| |
| --- | --- | --- |

Naeem A. Virk^a^, Aziz-ur-Rehman^a^**, Javed Iqbal^b^*, Tahir Ali Chohan^c^, Abdullah R. Alzahrani^d^, Talha Jawaid^e^, Zia Ur Rehman^f,g^, Abida Khan^h^

**SUPPORTING INFORMATION**


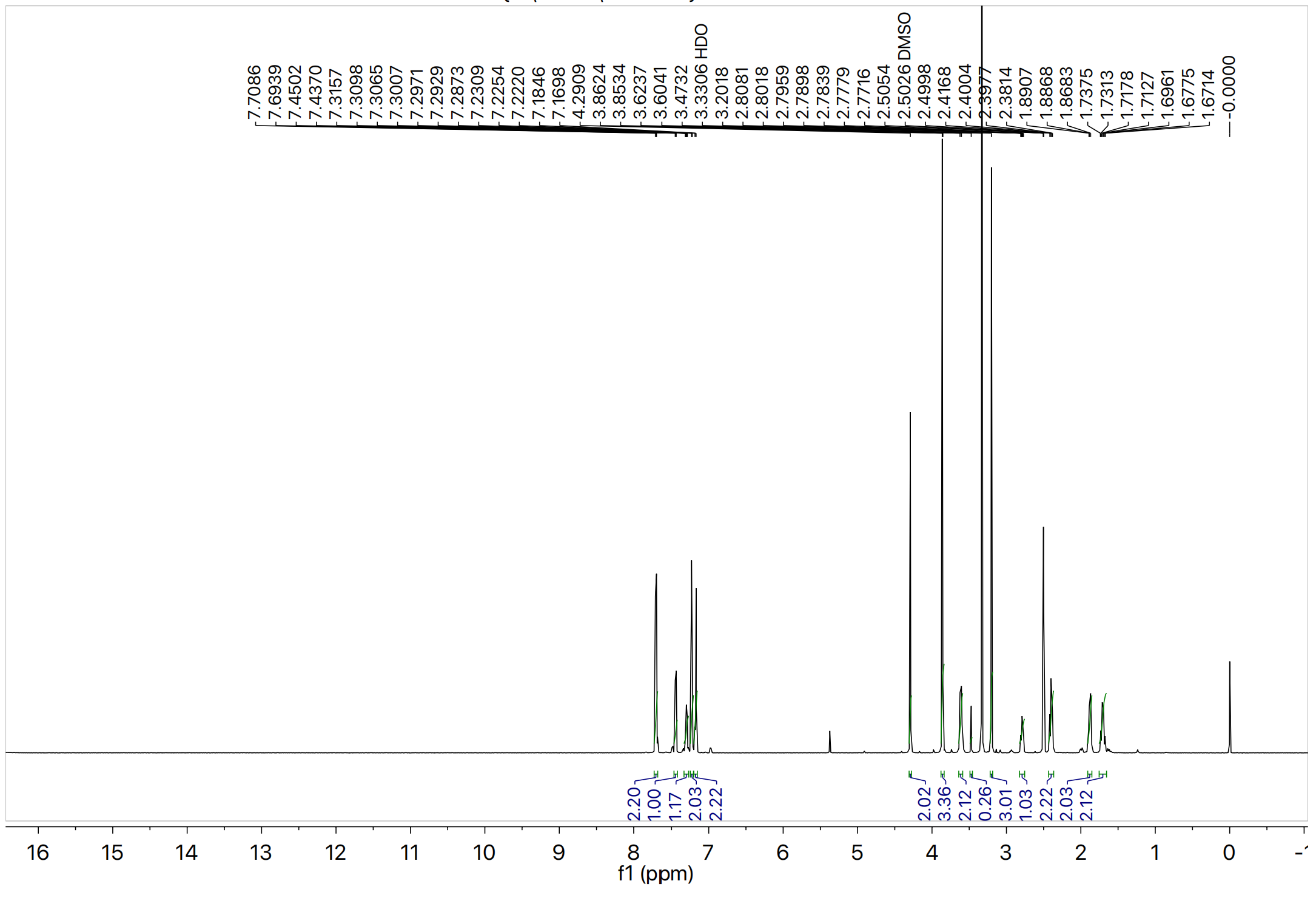


**S18 Fig.** ^1^HNMR spectrum of 3-[(3-bromobenzyl)thio]-5-{1-[(4-methoxypheneyl)sulfonyl]-4-piperidinyl}-4-methyl-4*H*-1,2,4-triazole **(7i)**

**(7j)**
